# Inhibiting LSD1 unlocks retinoid AP-1 programming to activate epithelial immunity and skin tumor suppression

**DOI:** 10.1172/JCI189044

**Published:** 2026-03-12

**Authors:** Nina Kuprasertkul, Alyssa F. Moore, Carina A. D’souza, Julia Chini, Eun-Kyung Ko, Sijia Huang, Shuo Zhang, Ashley S. Anderson, Shaun Egolf, Laura V. Pinheiro, Alison Jaccard, Claudia T. Magahis, Lydia Bao, Yann Aubert, Cyria Olingou, Stephen M. Prouty, Donna Brennan-Crispi, David A. Hill, John T. Seykora, Kathryn E. Wellen, Brian C. Capell

**Affiliations:** 1 Department of Dermatology and; 2 Penn Epigenetics Institute, University of Pennsylvania Perelman School of Medicine, Philadelphia, Pennsylvania, USA.; 3 Division of Allergy and Immunology, Children’s Hospital of Philadelphia, Philadelphia, Pennsylvania, USA.; 4 Department of Pediatrics,; 5 Penn Institute for Biomedical Informatics,; 6 Department of Cancer Biology,; 7 Abramson Family Cancer Research Institute, and; 8 Department of Genetics, University of Pennsylvania Perelman School of Medicine, Philadelphia, Pennsylvania, USA.

**Keywords:** Dermatology, Oncology, Cancer, Epigenetics, Skin

## Abstract

Lysine-specific demethylase 1 (LSD1; KDM1A) orchestrates context-dependent chromatin programs, yet its role in epithelial immunity remains largely unknown. Here, we identify LSD1 as a central brake on retinoid-driven and activator protein-1–driven (AP-1–driven) enhancer activation in epidermis and demonstrate that its inhibition induces antitumor immunity. Whereas epidermal LSD1 is required during development, acute loss or topical inhibition in adult skin was tolerated and triggered coordinated expression of retinoic acid signaling, lipid remodeling, and chemokine induction pathways. CUT&RUN profiling revealed that LSD1 occupies enhancer regions enriched for AP-1 motifs at retinoid metabolism, lipid homeostasis, and immune genes. LSD1 loss increased H3K4me1/2 and gene activation at these sites, licensing a poised AP-1–retinoid program. Single-cell spatial analyses showed that discrete keratinocyte subsets initiate retinoid signaling to recruit dendritic cells and activate CD4^+^ T cell responses. Topical LSD1 inhibition suppressed cutaneous squamous cell carcinoma in 2 models while amplifying keratinocyte–immune cell crosstalk. Functional perturbations revealed that retinoid signaling partially contributes to, whereas CD4^+^ T cells are essential for, tumor control. These findings define LSD1 as a master repressor of epithelial immune competence and nominate LSD1 inhibition as a therapeutic strategy to activate retinoid–AP-1 enhancer circuits and drive CD4-dependent tumor immunity in skin cancer.

## Abstract 2

This study reveals that an epigenetic regulator, while essential for skin development, can also be targeted to treat skin cancer through its ability to promote differentiation and immunosurveillance.

## Graphical abstract

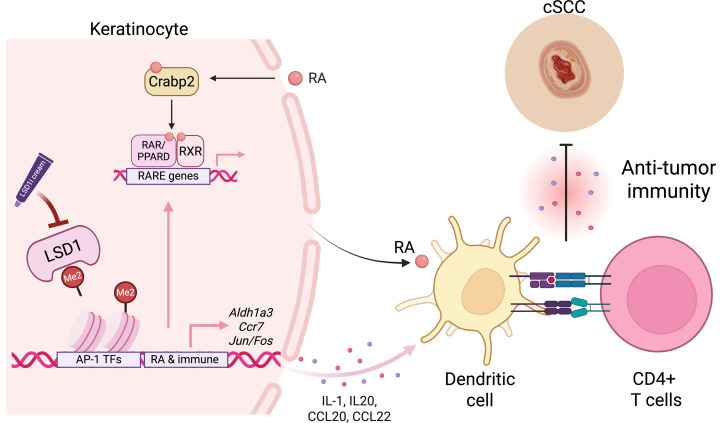

## Introduction

Lysine-specific demethylase 1 (LSD1; KDM1A) governs cell fate decisions underlying tissue development, differentiation, and homeostasis (1). The dynamic and reversible nature of LSD1 renders it ideal for therapeutic targeting, with LSD1 inhibitors potentiating immunotherapy approaches in breast, cervical, and colon cancer (2). By demethylating activating H3K4me1/2 marks at differentiation genes, LSD1 represses these programs and preserves progenitor-like fates (1). Yet, its role in coordinating differentiation and immune activation in skin is poorly understood.

The skin epidermis is a stratified epithelium that self-renews via keratinocyte basal stem cell differentiation into the terminal barrier (3). This barrier defends against pathogenic insults while maintaining immune homeostasis — a balance governed by keratinocytes, immune cells, and vitamin A–derived retinoids including retinoic acid (RA) (4). RA mediates keratinocyte differentiation and immune cell dynamics, with deficiencies precipitating marked immune dysfunction, underscoring the widespread clinical utility of topical retinoids (4–7). While RA can be immunosuppressive, it can also drive CD4^+^ Th1 and Th17 responses during inflammation (8–10). Retinoid signaling is governed by activator protein-1 (AP-1) transcription factors (TFs), master regulators of stress responses, inflammation, and tissue remodeling in the skin (11–13). Together, AP-1 factors and RA establish a network that preserves epidermal homeostasis while enabling rapid immune immobilization (14–16).

Yet, the epigenetic mechanisms coupling keratinocyte differentiation and retinoids to immune activation remain incompletely characterized, limiting our ability to harness RA’s immunomodulatory potential. Although LSD1 influences retinoid signaling in pancreatic development and leukemia treatment, its role in cutaneous retinoid pathways remains unexplored (17, 18). Here, we characterize LSD1’s context-dependent functions across skin development, homeostasis, and tumorigenesis. We discover that LSD1 loss activates retinoid metabolism and triggers dendritic cell (DC) and T cell infiltration. Genomic mapping reveals LSD1 occupancy at AP-1–enriched regions, with increases in H3K4me2 upon LSD1 loss at retinoid and pro-inflammatory loci. Single-cell spatial profiling identifies keratinocytes that acquire enhanced RA metabolism and DC-like signatures upon LSD1 depletion. We further define retinoid-dependent and -independent immune responses downstream of LSD1 inhibition, establishing RA as an early mediator of immune activation. Finally, we show that LSD1 inhibition harnesses these retinoid-immune networks to restrict cutaneous squamous cell carcinoma (cSCC) growth.

## Results

### LSD1 is required for embryonic epidermal development and barrier establishment.

**Figure 1:**
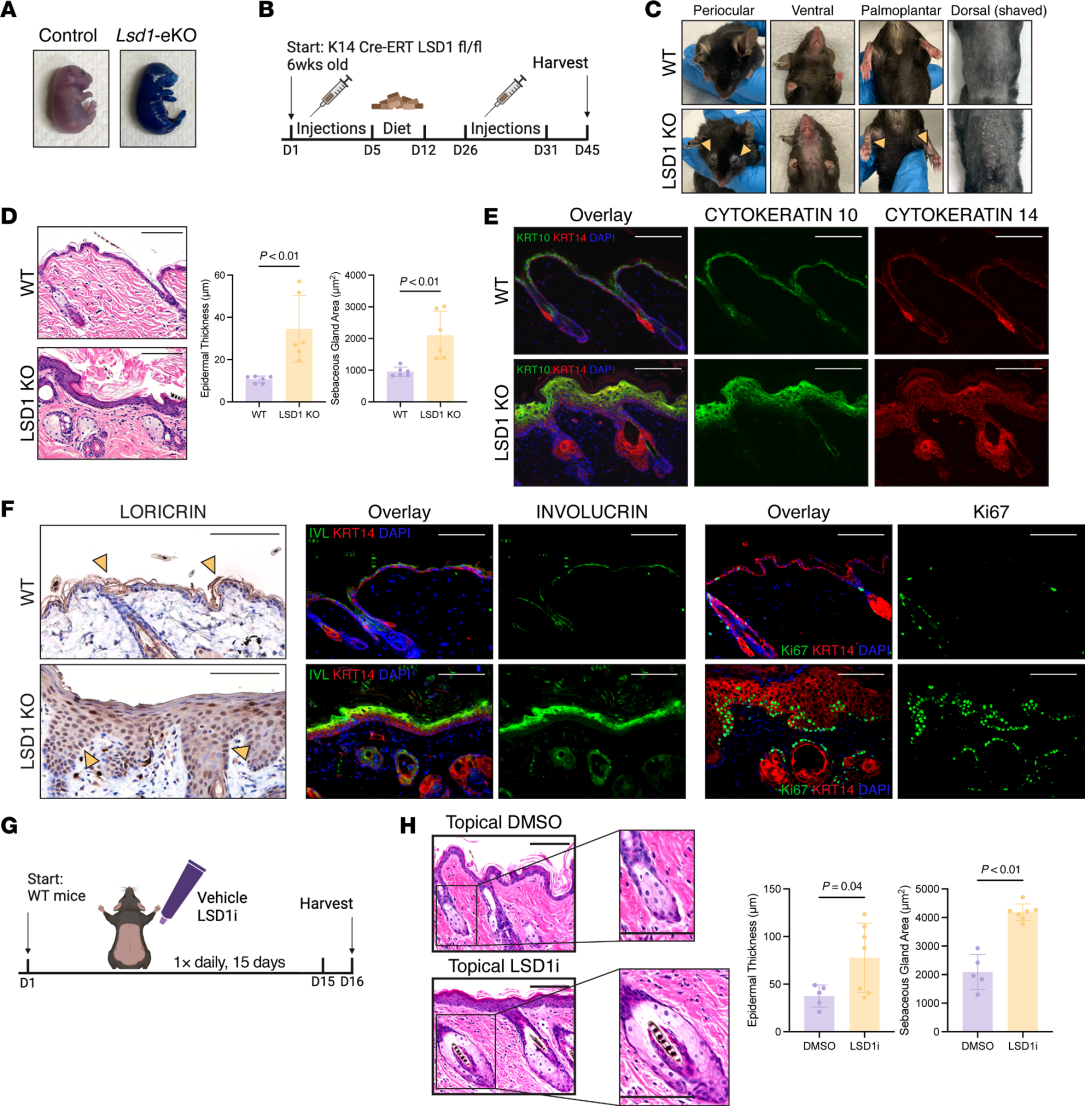
LSD1 is required for epidermal barrier development and maintenance of adult homeostasis. (**A**) Toluidine blue barrier assay of control and Lsd1-KO (Lsd1-eKO) embryos at E17.5. (**B**) Tamoxifen induction schematic for inducible *Lsd1* deletion in adult mice (LSD1-KO). (**C**) Gross phenotype of wild-type (WT) and LSD1-KO mice taken posteuthanasia at approximately 12 weeks old. Yellow arrows highlight phenotypes. (**D**) H&E of WT and LSD1-KO mice with quantification. (**E**) Immunofluorescence (IF) for KRT10 (green) in WT and LSD1-KO mice. KRT14 (red) and DAPI (blue). (**F**) Immunohistochemistry (IHC) for LOR (yellow arrows indicate staining) (left), IF for IVL (green) (middle); IF for Ki67 (green) (right) in WT and LSD1-KO mice. KRT14 (red) and DAPI (blue). (**G**) Topical LSD1 inhibition (LSD1i) schematic. (**H**) H&E of topical DMSO– or topical LSD1i–treated mice with quantification. Inset depicts sebaceous glands at same scale. All scale bars: 100 μm. Data represented as mean ± SD. Each dot represents an individual mouse: for **A**, *n* = 16 control and *n* = 3 Lsd1-eKO; for **C**, images representative of 4–5 independent experiments; for **D**, *n* = 6 mice per condition; for **E** and **F**, *n* = 3 mice per condition for staining; for **H**, *n* = 5–7 mice per condition. Two-tailed Student’s *t* test unless indicated.

To determine the function of LSD1 in skin, we generated mice with a constitutive epidermal specific deletion of *Lsd1* using a *Cytokeratin 14*-Cre (*Krt14*-Cre) driver to target epidermal basal stem cells (Lsd1-eKO). Lsd1-eKO mice were not recovered past E17.5 stemming from severe barrier failure, evidenced by gross skin abnormalities and dye penetration (Figure 1A and Supplemental Figure 1A; supplemental material available online with this article; https://doi.org/10.1172/JCI189044DS1). Despite normal embryonic weight, Lsd1-eKO mouse skin was markedly thinned, with diminished to completely absent development of suprabasal epidermal layers (Supplemental Figure 1, B–D). Loss of LSD1 was accompanied by global gains in H3K4me1 and H3K4me2, both of which showed restriction to the basal layer in controls (Supplemental Figure 1E). Despite these defects, we observed no changes in Ki67^+^ proliferating cells, TUNEL^+^ apoptotic cells, or expression of developmental TFs including p63 and KLF4 (Supplemental Figure 1, F–I). This suggested that the defects resulted from subtle transcriptional changes.

Single-cell spatial profiling with 10x Genomics Xenium on E17.5 control and Lsd1-eKO embryos revealed marked genotype-specific differences, most prominently within epidermal keratinocytes (Supplemental Figure 2, A and B, and Supplemental Table 1). As expected, Lsd1-eKO mice lacked an upper differentiated keratinocyte cluster (Supplemental Figure 2, A and B). Analysis of remaining basal layer cells (*Krt5*^+^*Col17a1*^+^) and suprabasal transitional cells (*Krt5*^+^*Krt1*^+^) (Supplemental Table 1) revealed that basal cells upregulated barrier establishment and keratinocyte differentiation genes, while transitional cells exhibited a metabolic differentiation signature, including fatty acid elongation, vitamin A (RA) response, and keratinization (Supplemental Figure 2, C and D). Developmental TFs (*Ovol2*, *Trp63*, *Snai2*, *Grhl3*) remained aberrantly active in transitional cells, suggesting failed transcriptional repression (Supplemental Figure 2E). Downregulation of cell cycle entry genes further implied premature exit from the proliferative state (Supplemental Figure 2E).

*Mki67* expression levels were decreased in Lsd1-eKO mouse transitional cells, uncovering layer-specific proliferative defects not captured by bulk staining (Supplemental Figure 2E). Regions lacking *Mki67* had accumulation of *Ovol2*, a master TF that promotes epithelial differentiation (19) (Supplemental Figure 2F). *Ovol2* expression was sparse in controls but was abundantly expressed throughout Lsd1-eKO epidermis (Supplemental Figure 2F). Thus, our data suggest that failure to repress developmental TFs in transitional cells, combined with premature activation of RA signaling, a critical developmental morphogen (20), collectively drives precocious differentiation and stratification defects.

### Maintenance of adult skin homeostasis is dependent on LSD1.

Adult tamoxifen-inducible KRT14-CreERT *Lsd1*-knockout mice (LSD1-KO mice) remained viable with minimal weight changes but displayed periocular and ventral alopecia, palmoplantar and dorsal scale, epidermal thickening, and sebaceous gland hyperplasia (Figure 1, B–D, and Supplemental Figure 3, A and B). These findings were not restricted to dorsal epithelium, as tongue epithelia of LSD1-KO mice also displayed hyperplasia (Supplemental Figure 3C). LSD1-KO mice showed gains in proliferation (Ki67), stemness (KRT14), and differentiation markers (INVOLUCRIN, LORICRIN, KRT10), suggestive of enhanced barrier formation (Figure 1, E and F). Mirroring embryonic findings, LSD1-KO mice also showed increased OVOL2-expressing cells (Supplemental Figure 3D). The contrasting phenotypes, developmental arrest versus hyperproliferation, likely stem from LSD1’s developmental stage–specific interactions with core TFs, including OVOL2.

To develop a therapeutic approach for targeting LSD1 in skin, we formulated a catalytic inhibitor of LSD1 (ORY-1001) (21) as a topical 0.000025% cream and applied LSD1i cream or vehicle to dorsal backs of WT mice for 12–15 days (Figure 1G). Topical LSD1i treatment was well tolerated, with mice developing dorsal scale and mild weight loss while retaining expression of LSD1 (Supplemental Figure 3, E–G). Consistent with genetic loss, topical LSD1i led to epidermal thickening, sebaceous gland hyperplasia, and increase in proliferation and stemness markers (Figure 1H and Supplemental Figure 3H). Given that ORY-1001 is in clinical trials (22), these results provided rationale to further investigate our topical LSD1i system for its clinical potential and as a more accessible mechanistic model.

### LSD1 regulates AP-1–driven immune and retinoid metabolism genes.

To uncover transcriptional changes downstream of LSD1 loss, we performed RNA-seq on LSD1-KO epidermis (Supplemental Figure 4A and Supplemental Table 2). ChEA3 analysis of 357 upregulated genes in LSD1-KO mice identified known LSD1 TF targets (*Ovol2*, *Grhl3*, *Znf750*, and *p63*) (Supplemental Figure 4B) (23). Gene Ontology (GO) analysis revealed retinoid metabolism as the top pathway, with upregulation of RA metabolic enzymes (*Rbp2*, *Crabp2*, *Aldh1a3*, *Dhrs9*) (Supplemental Figure 4, C and D). Topical LSD1i treatment induced 84 genes overlapping with those upregulated in LSD1-KO mice (Supplemental Figure 4E and Supplemental Table 3). These included keratinocyte differentiation genes, inflammatory mediators (*Il17a*, *S100a8*, *S100a9*, *Cd24a*), and RA signaling components (*Crabp2*, *Fabp5*, *Ppard*) (Supplemental Figure 4, E and F). Genes uniquely upregulated in LSD1-KO mice likely reflect LSD1’s scaffolding functions, preserved during catalytic inhibition but lost with protein deletion (24). Topical LSD1i treatment increased RA signaling marker CRABP2 protein levels, consistent with transcriptional changes (Supplemental Figure 4G). To assess off-target effects, we applied topical LSD1i to genetic LSD1-KO mice. Both treatments upregulated retinoid signaling, with topical LSD1i providing additional enhancement in knockout mice, likely reflecting inhibition in non-*Krt14*-expressing cells (Supplemental Figure 4H). Nevertheless, these data demonstrate that both genetic and pharmacological LSD1 inhibition activate RA metabolism.

**Figure 2:**
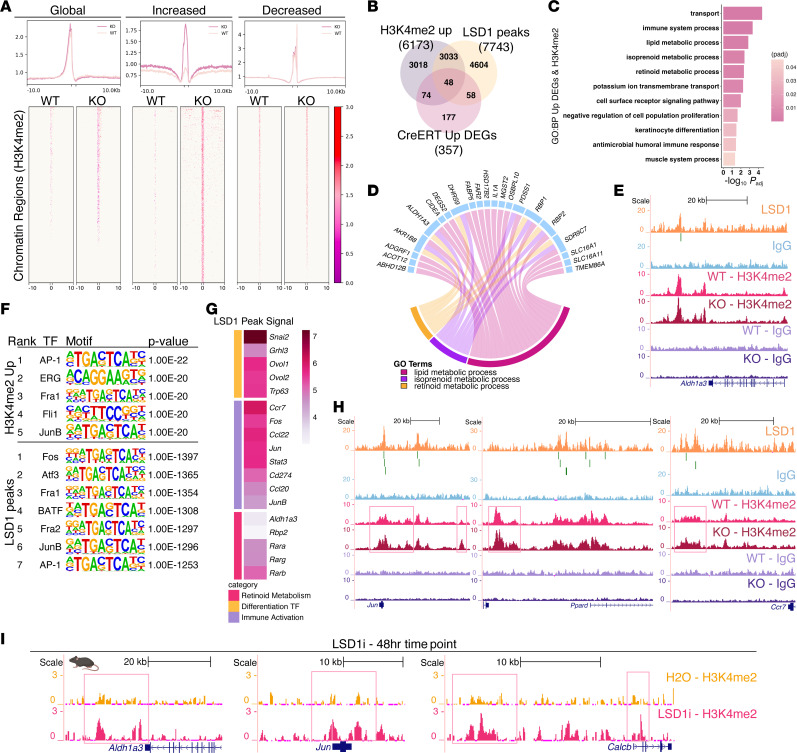
LSD1 regulates key immune-associated and retinoid metabolism genes. (**A**) Heatmap and corresponding metaplot of H3K4me2 signal per region spanning from –10 kb to 10 kb centered at peak center for denoted regions in LSD1-KO vs. WT mice as called by differential SICER analysis. (**B**) Overlap between genes associated with increased H3K4me2 regions in LSD1-KO vs. WT mice, upregulated genes in LSD1-KO vs. WT mice, and LSD1 peaks from CUT&RUN in WT mice. DEGs, differentially expressed genes. (**C**) GO analysis of overlap between upregulated genes and genes associated with increased H3K4me2 regions from Figure 2B. GO terms include immune system process (GO: 0002376), retinoid metabolism (GO: 0001523), and keratinocyte differentiation (GO: 0030216). (**D**) Chord plot of genes from retinoid-related GO terms in **C**. (**E**) LSD1 tracks in WT mice with H3K4me2 tracks in WT and LSD1-KO mice at *Aldh1a3*. (**F**) HOMER motif analysis of increased H3K4me2 peaks in LSD1-KO vs. WT mice and LSD1 peaks in WT mice. (**G**) Heatmap of averaged LSD1 peak signal at indicated genes. (**H**) LSD1 tracks in WT mice with H3K4me2 tracks in WT and LSD1-KO mice at *Jun*, *Ppard*, *Ccr7*. (**I**) H3K4me2 tracks in H_2_O- and LSD1i-treated mice for 48 hours at *Aldh1a3*, *Jun*, *Calcb*. For **A**–**H**, CUT&RUN data represent *n* = 2–3 replicates per condition, pooled from 4–5 mice per condition (see Methods); for **I**, CUT&RUN data represent *n* = 3 replicates per condition, pooled from 3 mice per condition (see Methods).

To elucidate the chromatin regulatory mechanisms underlying this response, we performed CUT&RUN (Cleavage Under Targets and Release Using Nuclease) to map LSD1 binding in WT mice and H3K4me2 distribution in WT and LSD1-KO mice. Although global H3K4me2 and H3K4me1 changes were undetectable by IHC (Supplemental Figure 5A), our prior work established that LSD1 inhibition preferentially increases H3K4me2 over H3K4me1 at specific loci (25). Analysis of H3K4me2-gained regions, likely direct consequences of lost demethylase activity, identified 6,173 associated genes enriched for T cell proliferation and interleukin signaling (Supplemental Table 4, Figure 2A, and Supplemental Figure 5B). Intersecting these with RNA-seq upregulated genes revealed 122 genes enriched for immune system processes, retinoid metabolism, and keratinocyte differentiation (Figure 2, B–D). Accordingly, we observed H3K4me2 accumulation at retinoid-synthesizing enzyme *Aldh1a3* (Figure 2E).

LSD1 localized predominantly to gene promoters and putative enhancers (downstream or distal intergenic regions) (Supplemental Figure 5C and Supplemental Table 5). Motif analysis of H3K4me2-gained peaks and LSD1-bound regions revealed profound enrichment for AP-1 TF binding sites known to orchestrate epithelial immunity, differentiation, and lipid metabolism (Figure 2F) (11–13, 26). LSD1 binding and H3K4me2 accumulation occurred at differentiation TFs (*Grhl3*, *Ovol2*, *Snai2*), retinoid and lipid metabolism mediators (*Ppard*, *Rara*), inflammatory AP-1 factors (*Jun*, *Fos*), and cytokine genes (*Ccr7*, *Il20*, *Il24*) (Figure 2, G and H, and Supplemental Figure 5, D and E). These data suggest that LSD1 acts as a master regulator coupling retinoid signaling to immune activation through control of specific transcriptional nodes. Supporting this model, we observed H3K4me2 accumulation at additional retinoid synthesis genes (*Aldh1a1*, *Dhrs9*) and target genes (*Calcb*) upon LSD1 deletion (Supplemental Figure 5F). H3K4me3 did not increase at these loci, confirming specificity for LSD1-catalyzed demethylation (Supplemental Figure 5G).

To distinguish direct chromatin effects from secondary responses, we performed CUT&RUN for H3K4me2 on epidermis treated with topical LSD1i for 48 hours. Early H3K4me2 accumulation occurred at retinoid genes (*Aldh1a3*, *Calcb*, *Jun*), with significant overlap between pharmacological and genetic perturbations in H3K4me2 distribution and gene expression changes (Figure 2I, Supplemental Figure 5H, and Supplemental Table 6). GO analysis confirmed enrichment for lipid/retinoid metabolism (*Rbp2*, *Dhrs9*, *Sdr9c7*), inflammatory response, and epidermal differentiation (Supplemental Figure 5H).

LSD1 chromatin immunoprecipitation with sequencing (ChIP-seq) in normal human epidermal keratinocytes (NHEKs) also validated conserved binding at key targets, including chemokine migration gene (*CCR7*), RA synthesis gene (*ALDH1A3*), and expected target genes (*SNAI2*, *OVOL2*) (Supplemental Table 7 and Supplemental Figure 5I).

These results highlight LSD1 as a chromatin gatekeeper directly repressing AP-1–driven retinoid synthesis and immune response genes. Under homeostatic conditions, LSD1 enforces silencing of immune genes through H3K4me2 demethylation. LSD1 loss redistributes H3K4me2, triggering derepression of an immune-primed keratinocyte state characterized by enhanced retinoid signaling (14).

### LSD1 loss leads keratinocytes to upregulate RA signaling.

**Figure 3:**
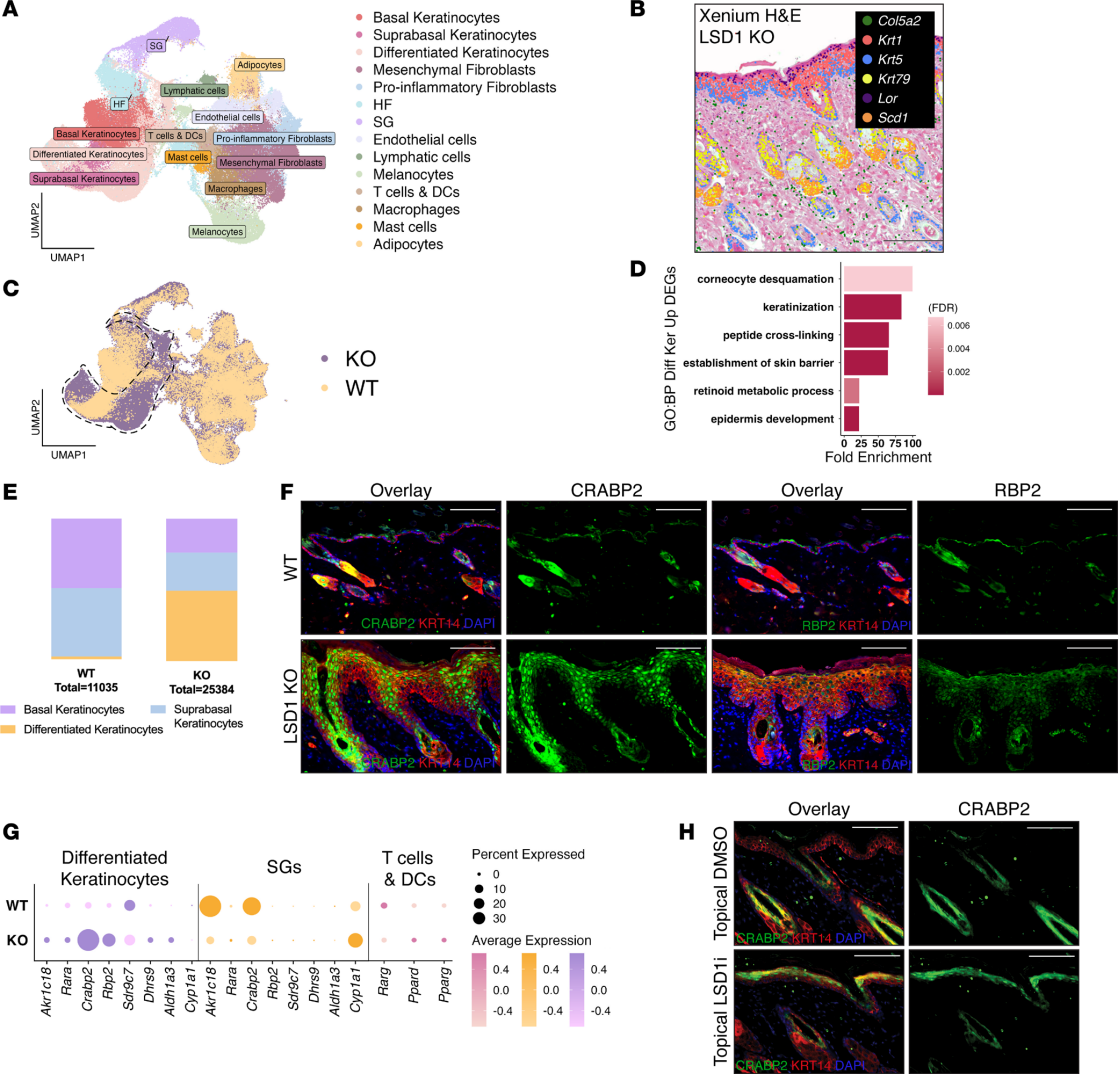
LSD1 loss directs keratinocytes to upregulate RA signaling. (**A**) Uniform manifold approximation and projection (UMAP) of merged WT and LSD1-KO Xenium data with cell types annotated. HF, hair follicle; SG, sebaceous gland. (**B**) H&E of LSD1-KO Xenium slide. Transcripts according to legend marked on slide. Representative image (*n* = 9) shown. (**C**) UMAP of WT and LSD1-KO Xenium data split by condition. Differentiated keratinocyte cluster approximated by dotted black line. (**D**) GO analysis of upregulated genes in LSD1-KO vs. WT mice from the differentiated keratinocytes cluster. GO terms include keratinocyte differentiation (GO: 0030216) and retinoic acid metabolism (GO: 0001523). (**E**) Proportion analysis of keratinocyte differentiation state from WT and LSD1-KO. (**F**) IF for CRABP2 (green, top) and RBP2 (green, bottom) in WT and LSD1-KO. KRT14 (red) and DAPI (blue). (**G**) Dot plot of RA genes across different clusters between WT and LSD1-KO. (**H**) IF for CRABP2 (green) in topical DMSO– or topical LSD1i–treated mice. KRT14 (red) and DAPI (blue). All scale bars: 100 μm. For **A**–**D** and **G**, *n* = 4–5 mice per condition; for **F** and **H**, *n* = 3 mice per condition for staining.

RA can be secreted by keratinocytes or immune cells in response to microenvironmental signals (16). To investigate how LSD1-mediated RA signaling governs keratinocyte–immune cell crosstalk, we performed Xenium profiling on the LSD1-KO skin, identifying 21 clusters representing 14 annotated cell types validated by correlating key marker transcripts with overlaid H&E staining morphology (Figure 3, A and B; Supplemental Figure 6A; and Supplemental Table 8). LSD1-KO mice displayed marked expansion of differentiated keratinocytes (*Lor*, *Sprr2a1*, *Flg2*) and enrichment of RA metabolism genes (Figure 3, C–E, and Supplemental Table 8). Specifically, genes for RA synthesis (*Dhrs9*, *Sdr9c7*, *Aldh1a3*), nuclear import (*Crabp2*), and downstream targets (*Akr1c18*, *Il1a*) were upregulated in both expression levels and proportion of cells, with the latter accounting for increased cell numbers in LSD1-KO mice (Supplemental Figure 6B).

RA carrier proteins CRABP2 and RBP2 showed increased levels by immunofluorescence and immunoblot upon LSD1 loss (Figure 3F and Supplemental Figure 6C). CRABP2, which facilitates nuclear import of RA (6), displayed primarily nuclear localization within the epidermis of LSD1-KO mice (Figure 3F). Xenium confirmed compartment-specific effects of LSD1 regulation with epidermal differentiated keratinocytes showing increased expression of RA metabolism genes, while sebaceous gland cells exhibited downregulation (Figure 3G). Topical LSD1 inhibition also recapitulated genetic knockout results, with increased expression of CRABP2 and RBP2 compared with vehicle (Figure 3H and Supplemental Figure 6D).

We next investigated whether LSD1 loss affected other cells in the skin microenvironment. T cells and DCs in LSD1-KO mice had altered expression of RA receptors, suggestive of response to changed RA levels (Figure 3G). Cytokine genes (*Tslp*, *Il1a*, *Il18*, *Ccr7*, *Ccl20*, *Ccl22*) showed both increased average expression and increased proportion of cells expressing them in LSD1-KO mice (Supplemental Figure 6E). Similarly, topical LSD1i upregulated genes involved in the inflammatory response (Supplemental Figure 6F). Both LSD1-KO and LSD1i-treated mice displayed increased CD3^+^ T cell staining, suggesting that LSD1 loss promotes T cell infiltration (Supplemental Figure 6G).

To determine whether these inflammatory signals originated from keratinocytes, we treated NHEKs with LSD1 inhibitor for 48 hours. This treatment upregulated myeloid-related genes (*CSF2*, *CCR7*) and pro-inflammatory mediators (*IL1B*) alongside known LSD1 targets (*SNAI2*) (Supplemental Figure 6H), demonstrating that keratinocytes directly produce inflammatory signals upon LSD1 inhibition.

### LSD1 inhibition initiates early activation of retinoid signaling followed by DC–T cell migration.

Given that LSD1 loss perturbed both RA and immune signaling, we sought to identify the sequence of events following LSD1 inhibition to differentiate early driver events from secondary compensatory responses.

Upon 48-hour LSD1i treatment, preceding histologic changes, *Aldh1a3* and *Crabp2* were significantly upregulated (Supplemental Figure 7, A and B). By day 5, CRABP2 protein was markedly elevated in LSD1i-treated epidermis, while CD3^+^ T cell infiltration remained absent (Supplemental Figure 7, C–E). Transcriptionally, we observed selective upregulation of RA metabolism genes while the expression of most cytokines, apart from *Ccl19*, remained unchanged (Supplemental Figure 7F).

The temporal separation between RA upregulation and immune infiltration suggested an intermediate step linking keratinocyte signaling to T cell recruitment. RA signaling elicits immune responses ranging from regulatory T cell induction to DC development and CD4^+^ T cell activation (9, 27–30). Thus, we profiled T cells and DCs from skin and draining lymph nodes (dLNs) by flow cytometry upon LSD1 inhibition (Supplemental Figure 8, A–C).

Following 8-day treatment with LSD1i, epidermal CD11b^–^ DCs decreased while accumulating in axillary dLNs (Supplemental Figure 9A). Since activated skin DCs migrate to dLNs to influence T cell responses (31, 32), we examined dLN immune activation. CD4^+^ T cell activation markers (ICOS, Ki67, T-BET) significantly increased within LSD1i-treated mouse dLNs (Supplemental Figure 9B). Total CD45^+^ immune cells remained unchanged in both compartments, suggesting that at this time point, LSD1i targets DCs and CD4^+^ T cells rather than inducing broad inflammation (Supplemental Figure 9C).

**Figure 4:**
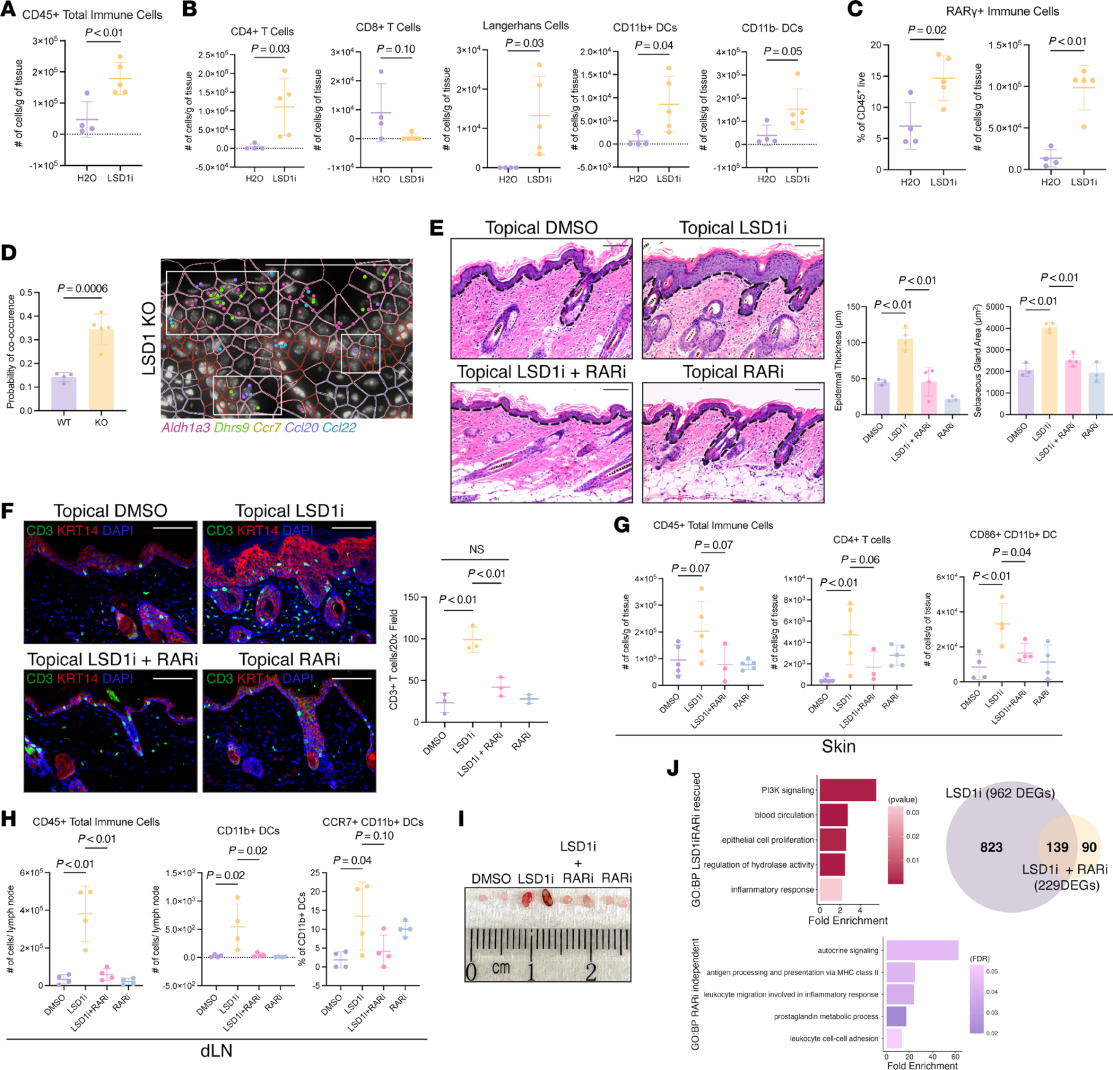
Epidermal phenotypes and immune infiltration upon LSD1 inhibition are dependent on retinoid signaling. (**A**) CD45^+^ live immune cells in epidermis of 12-day topical H_2_O- or LSD1i-treated mice. (**B**) Immune cell counts from 12-day-treated mice as in **A**. Multiple unpaired *t* tests. (**C**) RARγ^+^ immune cell frequency and counts in 12-day-treated mice as in **A**. (**D**) Xenium section in LSD1-KO mice with retinoid metabolism (*Aldh1a3*, *Dhrs9*) and immune (*Ccr7*, *Ccl20*, *Ccl22*) genes labeled. Boxes demonstrate localized signaling niches. Quantification on left. (**E**) H&E of mice treated with DMSO, LSD1i, LSD1i + RARi, and RARi with quantification; 1-way ANOVA. Dotted line indicates dermal-epidermal junction. (**F**) IF for CD3 (green) in dual LSD1i + RARi cohort. KRT14 (red) and DAPI (blue). Quantification on right; 1-way ANOVA. (**G**) CD45^+^ live immune cells and CD4^+^ T cells in whole skin of RARi cohort; 1-way ANOVA. (**H**) CD45^+^ live immune cells, CD11b^+^ DCs, and CCR7^+^CD11b^+^ DCs in skin-draining lymph nodes (dLNs) of RARi cohort; 1-way ANOVA. (**I**) Axillary lymph nodes from RARi cohort. (**J**) GO analysis of 823 rescued DEGs and 139 RARi-independent DEGs. GO terms include epithelial cell proliferation (GO: 0050678), inflammatory response (GO: 0006954), antigen processing and presentation via MHC class II (GO: 0019886), and leukocyte migration involved in inflammatory response (GO: 0002523) (left). Overlap between LSD1i DEGs vs. DMSO and LSD1i + RARi DEGs vs. DMSO (right). All scale bars: 100 μm. Data represented as mean ± SD. Each dot represents an individual mouse: for **A**–**D**, *n* = 4–5 mice per condition; for **E** and **J**, *n* = 3–4 mice per condition; for **F**, *n* = 3 mice per condition for staining; for **G**–**I**, *n* = 3–5 mice per condition. Two-tailed Student’s *t* test unless indicated.

By day 12, the initial lymph node immune response culminated in a return of immune cells to the skin. Total CD45^+^ immune cells, CD4^+^ T cells, and 3 DC subtypes were expanded in LSD1i-treated epidermis (Figure 4, A and B). Interestingly, while LSD1i-treated mice had visibly enlarged dLNs (Supplemental Figure 9D), total CD45^+^ immune cells, T cells, and DCs did not significantly change despite trending increases (Supplemental Figure 9E). Moreover, CD11b^–^ DCs in the dLNs showed decreased checkpoint (CTLA-4, PD-1, and PD-L2) and activation (ICOS) markers (Supplemental Figure 9F). These observations suggest that LSD1i treatment initiates early RA signaling and DC migration to dLNs that subsequently mount a T cell response back in the skin.

### LSD1i drives retinoid-dependent and -independent epidermal and immune responses.

We then investigated whether immune responses were a functional consequence of RA signaling, if bidirectional crosstalk was occurring, and how LSD1 factored into these interactions. We first assessed whether T cell recruitment to the skin was a downstream by-product or actively mediated homeostasis, as suggested by previous studies (33). CD4^+^/CD8^+^ T cell depletion with LSD1i did not alter weight loss, gross skin phenotype, or upregulation of retinoid genes observed with LSD1i treatment alone (Supplemental Figure 10, A–E). However, T cell depletion partially rescued epidermal thickness without affecting sebaceous gland hyperplasia (Supplemental Figure 10F), indicating that T cells contribute to but do not primarily drive LSD1i-induced phenotypes.

We noted increased recruitment of RARγ^+^ immune cells in LSD1i-treated mice, suggesting enhanced immune cell responsiveness to RA signaling (Figure 4C). In support of this, spatial transcriptomics revealed that RA metabolism and immune genes were coexpressed within the same cells or niches at significantly higher frequency in LSD1-KO mice (Figure 4D and Supplemental Figure 10, G and H), highlighting intimate crosstalk between RA and immune pathways.

To test whether RA signaling was necessary to drive the skin immune response, we employed the pan-RA receptor antagonist (AGN193109, RARi) alongside topical LSD1i (Supplemental Figure 10I). Concomitant treatment with RARi reversed LSD1i-induced epidermal thickening, sebaceous gland hyperplasia, CD3^+^ T cell infiltration, and gross skin changes (Figure 4, E and F, and Supplemental Figure 10I). Flow cytometry of whole skin confirmed RARi reversal of total CD45^+^ and CD4^+^ T cell infiltration as well as proportion of activated CD86^+^CD11b^+^ DCs (Figure 4G). RARi strongly prevented a global inflammatory response in skin dLNs, reflected in CCR7^+^ DC migration (Figure 4H), and gross phenotype of the dLNs (Figure 4I). Intriguingly, LSD1i-induced IL17^+^CD4^+^ T cell expansion in the skin persisted despite RARi, revealing retinoid-independent responses (Supplemental Figure 10J).

RNA-seq showed that while LSD1i alone altered 962 genes, dual treatment with LSD1i and RARi reduced this to 229 (Figure 4J and Supplemental Table 9). The 823 genes rescued with RARi treatment included epithelial cell proliferation and inflammatory response genes (Figure 4J). Genes independent of RARi included those involved in antigen processing and leukocyte migration (Figure 4J). Collectively, these data indicate that LSD1 inhibition activates RA-dependent pathways that drive major phenotypes and parallel retinoid-independent pathways.

### Topical LSD1i restrains tumor growth in cSCC models.

Retinoids have shown therapeutic potential for leukemia and for enhancing antitumor CD8^+^ T cell function (34, 35). However, in cSCC, clinical trials have yielded inconsistent results despite promising preclinical studies, prompting proposals for therapies to improve retinoid responsiveness (5, 36).

Concurrently, LSD1 inhibitors have gained traction in oncology, due to synergy with PD-1–blocking immunotherapies (2). Yet, while anti–PD-1 approaches were FDA-approved in 2018 and 2020 for cSCC, approximately half of all patients do not respond (37). We thus leveraged LSD1 inhibition to potentiate immune and retinoid signaling in 2 models of cSCC.

**Figure 5:**
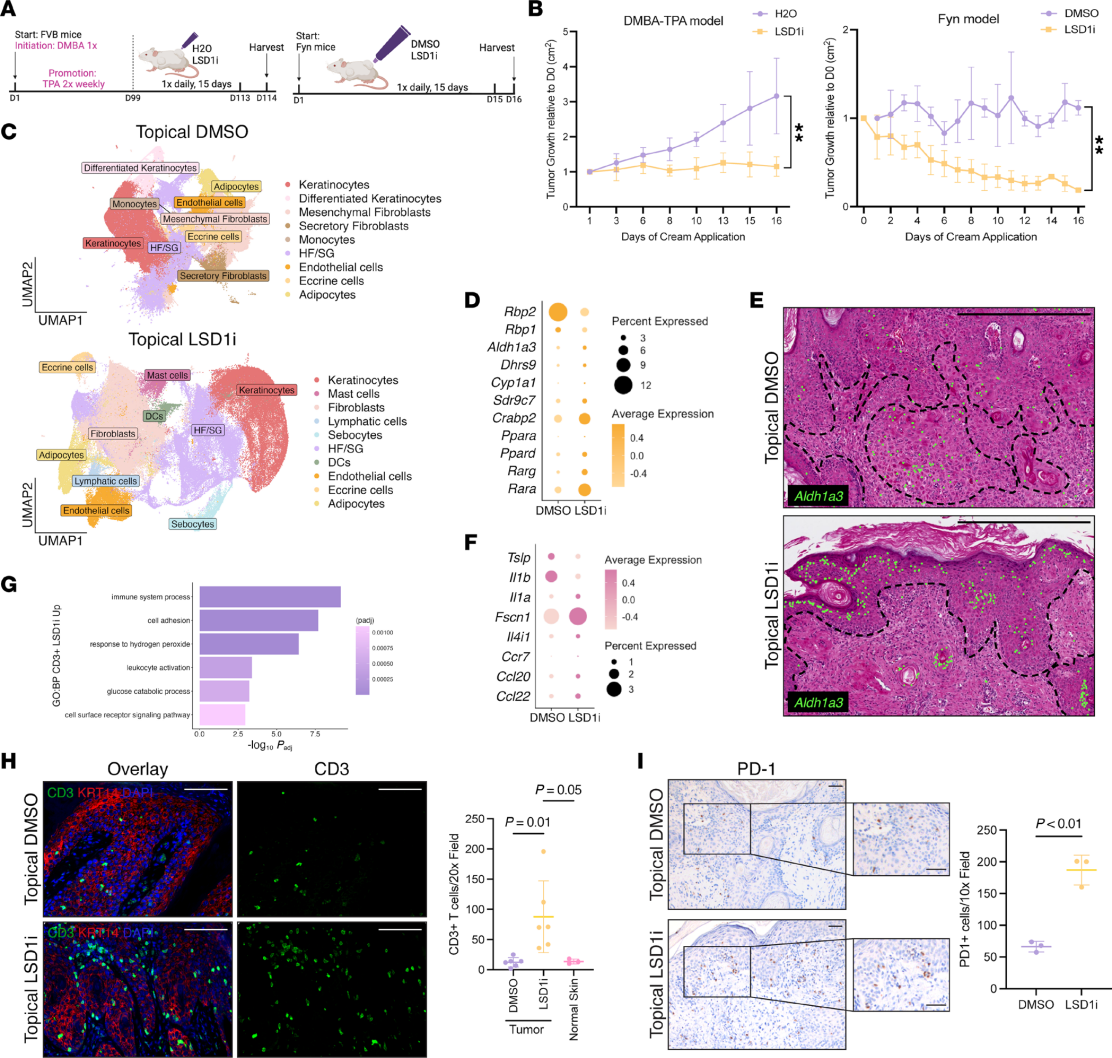
LSD1 inhibition modulates retinoid-immune pathways and restrains tumor growth. (**A**) Schematic for cSCC induction by chemical methods (DMBA-TPA, left) or genetic methods (Fyn mice, right). (**B**) Tumor area growth in DMBA-TPA (left) or Fyn model (right); multiple unpaired *t* tests (*P* value = *t* test on last day). (**C**) UMAP from Xenium data in topical LSD1i- or DMSO-treated Fyn tumors. (**D**) Dot plot with RA genes from Xenium in LSD1i- or DMSO-treated Fyn tumors. (**E**) Xenium H&E sections from topical LSD1i- or DMSO-treated tumors, *Aldh1a3* (green) labeled. Dotted line indicates approximation of dermal-epidermal junction or tumor area. Scale bar: 500 μm. (**F**) Dot plot with immune genes from Xenium in LSD1i- or DMSO-treated Fyn tumors. (**G**) GO analysis of genes upregulated in CD3^+^ T cells in LSD1i- vs. DMSO-treated Fyn tumors. GO terms include leukocyte activation (GO: 0045321) and glucose catabolism (GO: 0006007). (**H**) IF for CD3 (green) in Fyn tumor cohort. KRT14 (red) and DAPI (blue). Quantification on right; 1-way ANOVA. (**I**) IHC for PD-1 in Fyn tumor cohort. Quantification on right; 1-way ANOVA. Scale bars: 100 μm unless indicated. Data represented as mean ± SD. Each dot represents an individual mouse for bar graphs: for **B**, *n* = 4–5 mice per condition in DMBA-TPA model and *n* = 3–4 mice per condition in Fyn model; for **C**–**G**, *n* = 3 mice per condition; for **H**, *n* = 6 tumors across 3–4 mice per condition, *n* = 3 mice for normal skin for staining; for **I**, *n* = 3 mice per condition for staining. **P < 0.005.

First, we induced cSCCs through multistage chemical carcinogenesis with mutagenic 7,12-dimethylbenz[a]anthracene (DMBA) followed by tumor-promoting agent 12-*O*-tetradecanoylphorbol-13-acetate (TPA) for 12 weeks (38). Upon papilloma or cSCC development, mice received topical LSD1i or vehicle daily for 15 days (Figure 5A). To complement this model, we used Krt14-Y528 FYN mice, which develop cSCCs by 7–8 weeks of age through oncogenic SRC kinase activation (Figure 5A) (39). This dual model allows for assessment of efficacy across distinct oncogenic landscapes. DMBA-TPA tumors are driven by *Hras* mutations alongside Wnt and pro-tumor inflammatory activation (38). Meanwhile, FYN mice show enhanced ERK1/2 signaling with suppression of Notch1 and p53 (39). Despite these divergent mechanisms, topical LSD1i significantly restricted tumor growth in both models (Figure 5B). DMBA-TPA–treated mice showed minimal weight changes or adverse effects while LSD1i-treated FYN tumors demonstrated marked histological improvement (Supplemental Figure 11, A–D). Tumor regression occurred without dramatic changes in cell proliferation or death as indicated by Ki67^+^ and TUNEL staining, respectively (Supplemental Figure 11, E and F).

To identify cell types underlying tumor regression, we performed Xenium spatial transcriptomics, which revealed a separate cluster of DCs (*Fscn1*, *Il4i1*, *H2-K1*, *Ccr7*, *Ccl22*) in LSD1i-treated tumors (Figure 5C, Supplemental Figure 12A, and Supplemental Table 10). LSD1i-treated tumors also had increased proportions of cells upregulating genes involved in RA synthesis (*Aldh1a3*, *Dhrs9*, *Sdr9c7*), nuclear import (*Crabp2*), and nuclear receptors (*Ppara*, *Ppard*, *Rarg*, *Rara*) (Figure 5D and Supplemental Figure 12B). Intriguingly, *Aldh1a3*-expressing cells, initially diffuse, redistributed to upper differentiated epidermal layers following LSD1i treatment (Figure 5E and Supplemental Figure 12C), suggesting that LSD1 inhibition not only enhances retinoid metabolism but also restores tissue architecture.

Consistent with our homeostatic studies, increased retinoid metabolism correlated with expansion of DCs and T cells. *Ccr7*-expressing cells markedly increased in epidermal and dermal LSD1i-treated tumor regions (Supplemental Figure 12D). DC genes (*Fscn1*, *Il4i1*, *Ccr7*, *Ccl22*), MHC-II gene (*H2-K1*), cytokine gene (*Il1a*), and T cell genes (*Cd3e*, *Cd3g*) increased in LSD1i-treated tumors (Figure 5F and Supplemental Figure 12, E and F). *Cd8a* expression remained unchanged, consistent with CD4^+^ T cell–specific effects (Supplemental Figure 12G). Despite this, T cells in LSD1i-treated tumors had increased expression of leukocyte activation and glucose catabolism genes, a metabolic feature of activated T cells (Figure 5G and Supplemental Table 10). Supporting a potential synergy with immunotherapy, LSD1i-treated tumors also displayed increased CD3^+^ and PD-1^+^ cell infiltration (Figure 5, H and I).

### LSD1i-mediated tumor suppression requires coordinated retinoid and immune pathway activation.

**Figure 6:**
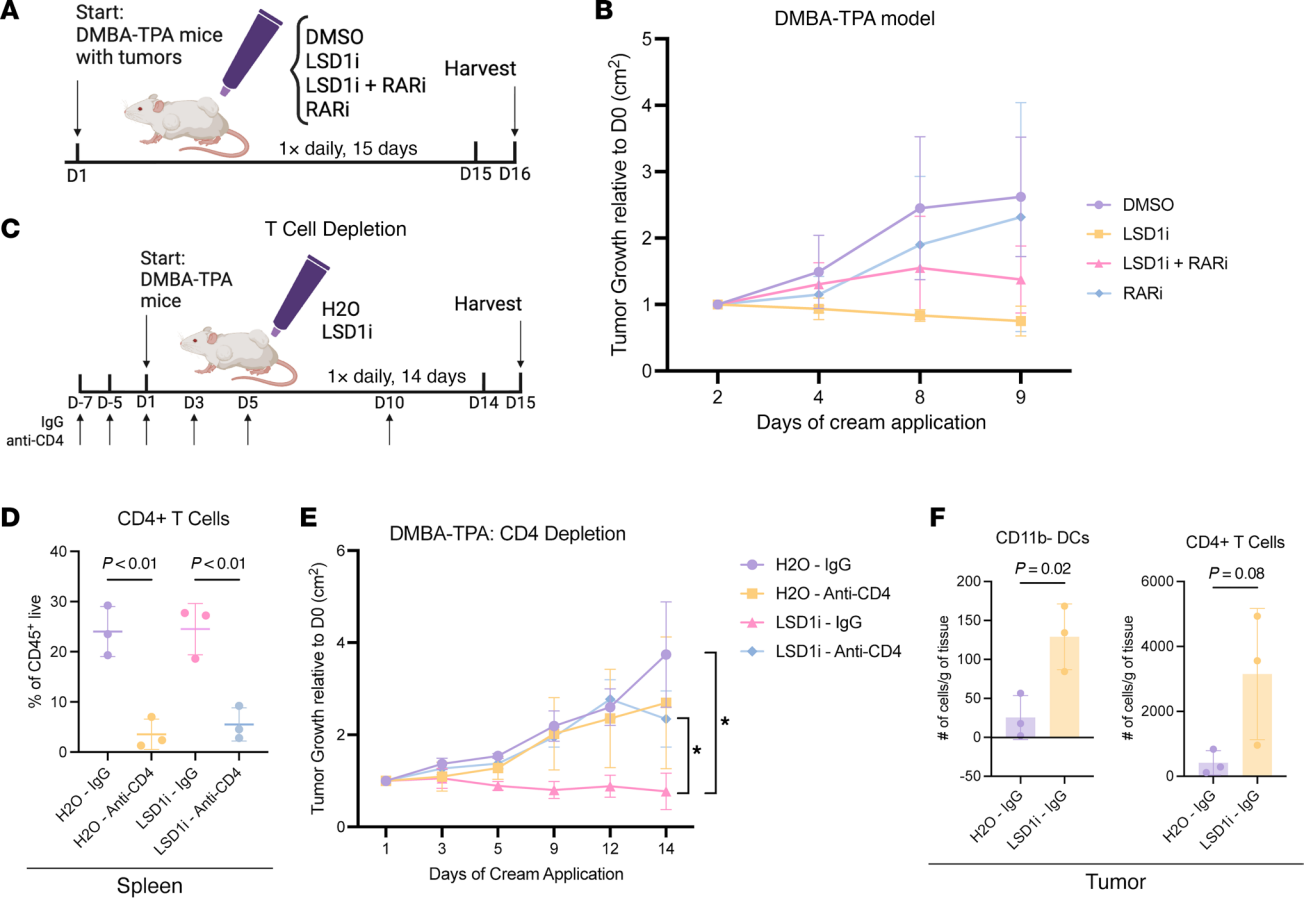
LSD1i tumor growth restriction requires retinoid-immune signaling. (**A**) Schematic for RARi rescue study of cSCC lesions. (**B**) Tumor area growth in RARi cohort; multiple unpaired *t* tests (*P* value represents *t* test on last day). (**C**) Schematic for CD4^+^ T cell depletion in cSCC lesions followed by H_2_O or LSD1i treatment. (**D**) Confirmation of CD4^+^ T cell depletion in spleen; 1-way ANOVA. (**E**) Tumor area growth in CD4^+^ T cell–depleted cohort; multiple unpaired *t* tests (*P* value represents *t* test on last day). (**F**) CD11b^–^ DC and CD4^+^ T cell counts in tumors from H_2_O- or LSD1i-treated mice (data taken from IgG controls of CD4^+^ T cell–depleted cohort); Two-tailed Student’s *t* test. Data represented as mean ± SD. Each dot represents an individual mouse for bar graphs and scatterplots: for **B**, *n* = 4 mice per condition; for **D**–**F**, *n* = 3 mice per condition. *P < 0.05.

We last investigated the functional requirements for LSD1i-mediated tumor suppression with rescue experiments in the DMBA-TPA model (Figure 6A). Administration of RARi partially rescued tumor growth restriction induced by LSD1i (Figure 6B). In contrast, CD4^+^ T cell depletion abolished tumor growth restriction effects of LSD1i, indicating that immune-mediated mechanisms play a predominant role compared with retinoid signaling (Figure 6, C–E). Indeed, flow cytometry revealed that LSD1 inhibition resulted in significant and trending increases of DCs and CD4^+^ T cells, respectively, within tumors (Figure 6F). Thus, while concurrent derepression of retinoid signaling and immune pathways contributes to tumor growth restriction, the immune component appears to be the critical mediator. Overall, our findings suggest that LSD1 inhibitors could enhance the efficacy of existing immunotherapies while overcoming retinoid resistance mechanisms in cutaneous malignancies.

## Discussion

Retinoids are versatile signaling metabolites implicated in keratinocyte differentiation, hair follicle lineage plasticity, wound healing, and immune responses (7). Yet, regulators connecting retinoid signaling to skin immunity remain incompletely understood. Here we show that LSD1 acts as an epigenetic switch controlling retinoid metabolism and inflammation, with implications for epidermal homeostasis and cancer progression.

Loss of just LSD1 was sufficient to derail embryonic epidermal barrier development through aberrant upregulation of developmental TFs (*Ovol2*, *Grhl3*) in basal and transitional keratinocytes. The upregulation of *Ovol2*, a known LSD1 target whose overexpression in basal keratinocytes drives precocious differentiation (19), suggests that LSD1 maintains developmental competence by preventing premature differentiation.

In adult mice, single-cell spatial transcriptomics demonstrated how LSD1 loss drove keratinocytes to upregulate retinoid metabolism, creating localized niches with enhanced coexpression of retinoid and immune genes. The compartment-specific effects, with keratinocytes increasing RA production while sebaceous glands show the opposite, reflects LSD1’s role in fine-tuning local retinoid availability. Whether epidermal derepression of *Ppard* and retinoid signaling leads to sequestering of retinoid precursors that drives secondary downregulation of retinoid synthesis genes in sebaceous glands and releases sebocytes from retinoid-mediated growth suppression, resulting in hyperplasia, warrants future investigation (40).

RARi reversal of LSD1 inhibition effects confirms the central role of retinoid signaling in our phenotypes. Indeed, the expansion of DCs and CD4^+^ T cells upon LSD1 inhibition aligns with RA’s known immunomodulatory role (9, 27, 41, 42). The partial rescue of epidermal thickness with CD4^+^/CD8^+^ T cell depletion underscores the complex immune community in skin, where innate immune cells or keratinocytes are additional players. We find that LSD1 represses a *Ccr7* signaling axis in keratinocytes, highlighting keratinocytes as components of structural immunity given that CCR7 is normally expressed on DCs to mediate T cell migration to lymph nodes (31, 43). RARi also prevented the migration of CCR7^+^ DCs to lymph nodes, further supporting LSD1-mediated retinoid regulation of DC migration. The persistence of IL17^+^CD4^+^ T cells, despite RAR blockade, reveals parallel retinoid-independent immune pathways mediated by LSD1. Indeed, recent work has highlighted LSD1’s role in regulating skin innate immunity beyond retinoid signaling (44). Our observation of upregulated antimicrobial peptide genes (*S100a8*, *S100a9*), Th17 response genes (*Il17a*), and pathogen-specific pattern recognition receptors (*Tlr3*, *Ddx58*) upon LSD1 loss (Supplemental Figure 6F) aligns with these findings (44), demonstrating that the TF ZNF750 interacts with LSD1 to repress pathogen-specific innate immunity. We extend these findings by uncovering which LSD1-mediated immune effects are RA responsive versus RA independent.

Our chromatin profiling mechanistically revealed an inflammatory network governed by LSD1 including AP-1–driven epithelial immune genes (*Jun*, *Fos*), as well as inflammatory TFs and cytokines (*Stat3*, *Ccl20*, *Ccl22*). These findings echo observations in intestinal epithelium, where LSD1 regulated immunity through chemokine expression (45), though intestinal development remained unaffected by LSD1 loss (45), in contrast with skin. Given the emerging roles of JUN and FOS in mediating inflammatory memory downstream of skin insult, future investigations could explore how LSD1 mediates these responses through retinoid activation of DC/T cell signaling (11).

Finally, LSD1 inhibition is relevant in cSCC, where immunosuppression is a major risk factor and retinoids have yielded mixed responses (46–50). Topical LSD1i suppressed tumor growth in 2 models, demonstrating applicability across cSCC subtypes. While blocking retinoid signaling partly prevented LSD1i growth restriction, CD4^+^ T cell depletion returned responses to baseline, revealing LSD1i’s retinoid-independent immunomodulatory effects. This dual mechanism suggests that LSD1i may overcome the limited efficacy of retinoid monotherapy.

Thus, from embryonic development through cancer progression, LSD1 functions as a master regulator of skin biology through coordinated control of differentiation, retinoid, and immune signaling networks. Topical epigenetic therapies could present a powerful and easily applicable tool for rewiring immune responses in skin cancer, avoiding drawbacks associated with surgical excision or systemic chemotherapy.

## Methods

### Sex as a biological variable.

Keratinocytes were obtained from neonatal foreskin, requiring use of male donors for cell culture. Our study utilized both male and female mice in comparable ratios and reported similar findings for both.

### Murine models.

Mice were on a mixed C57BL/6 (LSD1 mice) or FVB (FYN mice) background on a 12-hour light/dark cycle and given food/water ad libitum. For LSD1 studies, mice carrying *loxP* sites flanking exons 5 and 6 of *Kdm1a* (Jackson Laboratory 023969; *Lsd1*^fl/fl^) were crossed with Krt14-Cre transgenic mice (Jackson Laboratory 018964). Krt14-Cre: *Lsd1*^fl/fl^ (LSD1-eKO) were considered knockouts. Age- and sex-matched littermates lacking Krt14-Cre and homozygous for *Lsd1* alleles were controls. For inducible knockouts, *Lsd1*^fl/fl^ mice were crossed with Krt14-CreERT mice (Jackson Laboratory 005107), generating Krt14-CreERT: *Lsd1*^fl/fl^ mice (LSD1-KO). For knockout, mice 6–7 weeks of age were injected intraperitoneally (i.p.) with tamoxifen (Sigma-Aldrich T5648-1G) resuspended in corn oil (Sigma-Aldrich C8267) at 100 mg/kg dose for 5 days. Mice were then given tamoxifen diet for 7 days followed by 2-week monitoring and subsequent i.p. injections for 5 days, then harvested after 2 weeks. Genotype controls and corn oil (vehicle) controls were used. For topical studies, WT mixed C57BL/6/B129 mice (from crosses above) or WT C57BL/6 mice (Jackson Laboratory 000664) were used, with the same background maintained within cohorts. WT FVB mice (Jackson Laboratory 001800) were used for DMBA-TPA models. Fyn mice were from the Seykora laboratory (39). Tail snip genotyping was performed with a Phire Tissue Direct PCR Kit (Thermo Fisher Scientific F-170L) with the following: Cre-F (5′ GAACCTGATGGACATGTTCAGG 3′), Cre-R (5′ AGTGCGTTCGAACGCTAGAGCCTGT 3′), Lsd1-F (5′ GCTGGATTGAGTTGGTTGTG 3′), Lsd1-R (5′ CTGCTCCTGAAAGACCTGCT 3′), Krt14-F (5′ AACGTGCTGGTTATTGTGCTG 3′), Fyn-R (5′ TTCCGTCCGTGCTTCATAGT 3′) (51).

### Embryo timed breeding and barrier dye assay.

Soiled male (Krt14-Cre: *Lsd1*^+/fl^) bedding was placed in female (Krt14-Cre negative: *Lsd1*^fl/fl^) cages for 3–4 days prior to mating. Male and female mice were mated for 1 night (14–15 hours) and males removed after (E0.5). Pregnant mice were harvested at E17.5. Embryos were dissected, weighed, and anesthetized with cold 100% methanol for 3 minutes. Embryos were soaked in 100%, 75%, 50%, 25%, 25%, 50%, 75%, and 100% methanol each for 1 minute, stained with 0.1% toluidine blue for 5 minutes, washed with 1× cold PBS, and imaged.

### Histology.

Isolated embryos were decapitated for euthanasia and dorsal whole skin dissected. For adult mice, upper dorsal or ventral whole skin was dissected. Tissues were fixed in 4% paraformaldehyde (Thermo Fisher Scientific J19943-K2) overnight at 4°C. Tissues were processed into formalin-fixed, paraffin-embedded (FFPE) blocks by Core A (Cutaneous Phenomics and Transcriptomics) of the Penn Skin Biology and Diseases Resource-based Center (SBDRC). H&E staining was performed by the SBDRC (including after Xenium H&E staining) and imaged with a Leica DM6B microscope. Histology was quantified using length and area on Fiji/ImageJ (v2.14.0). Scale was set to a known pixel length equals 100 μM according to scale bars on images.

### Murine topical studies.

WT mouse dorsal backs were shaved 1 day prior. Cetaphil cream was mixed with drug or vehicle and applied to the upper dorsal back for the indicated time. Skin was harvested 24 hours after the last treatment. For LSD1 inhibition, ORY1001 (AstaTech 40826 or SelleckChem S7795) was dissolved in DMSO or H_2_O, then mixed with cream to generate 0.000025% cream (w/v). For RAR inhibition, AGN193109 (Sigma-Aldrich SML2034) was dissolved in DMSO and mixed with cream to generate 0.000025% cream. For tumor studies, ORY1001 or DMSO (FYN model)/H_2_O (DMBA-TPA model) was applied to tumors and covered with 100 mg cream daily for a dose of 0.00005% cream. Tumors < 0.3 cm^2^ starting area were excluded. Tumor area was calculated using area selection in Fiji/ImageJ (v2.14.0), normalized to areas on day 0, and graphed as tumor growth per day (tumor area day X/tumor area day 0).

### DMBA-TPA chemical carcinogenesis.

As previously described (38), WT FVB mice 7–10 weeks of age were treated once with 100 nmol DMBA (SelleckChem E1022) dissolved in acetone (100 nmol/0.2 mL). Afterward, mice were treated twice weekly with 8.5 nmol tumor-promoting agent TPA (Cell Signaling Technology 4174) dissolved in acetone (8.5 nmol/0.2 mL). Mice were shaved as needed. Tumor-bearing mice (>0.3 cm^2^ starting area, about 12 weeks postinitiation) were randomized to treatment with either H_2_O or ORY1001. TPA was stopped upon treatment.

### Murine RNA and protein extraction.

Mouse skin was shaved (RNA) or shaved and treated with Nair (Church & Dwight) to completely remove all remaining hair (protein), dissected, and underlying fat pad scraped off. Tissue was floated dermis-side down in 1× dispase (5 U/mL; Corning 354235) in PBS for 50 minutes at 37°C and epidermis dissociated by scalpel. For RNA, the epidermis was flash-frozen in 1 mL TRIzol (Invitrogen 10296028). Tissue was homogenized with MP Biomedical Homogenizer and RNA extracted with RNeasy Kit (QIAGEN 74104). For protein, the epidermis was scraped into cold PBS and centrifuged at 4°C for 10 minutes at 1,188*g*. A size-dependent volume of protein lysis buffer (Cell Signaling Technology 9803) was added. The mixture was homogenized, sonicated, rotated at 4°C for 10 minutes, and then centrifuged at 4°C for 10 minutes at full speed. The supernatant was stored at –80°C.

### NHEK culture.

Proliferating primary NHEKs were isolated from deidentified discarded neonatal foreskin donors provided by the Penn SBDRC. NHEKs were cultured in a sterile-filtered 50:50 mix of 1× keratinocyte serum-free medium (Gibco 17005042) supplemented with human recombinant epidermal growth factor (Gibco 10450-013) and bovine pituitary extract (Gibco 13028-014) combined with Medium 154 (Gibco M154500) supplemented with human keratinocyte growth supplement (Gibco S10015) and 1% 10,000 U/mL penicillin-streptomycin at 37°C with 5% CO_2_. NHEKs were maintained at 50%–70% confluence and passages 5–7 for experiments.

### Quantitative RT-PCR.

A total of 1,000 μg of RNA was used for cDNA using a High-Capacity cDNA Reverse Transcription Kit (Applied Biosystems 4368814). qPCR was performed with PowerSYBER Green PCR Master Mix (Applied Biosystems 4367659), diluted cDNA, and 10 μM forward and reverse primers. The qPCR was run on a ViiA 7 Real-Time PCR System (Applied Biosystems) using ΔΔCt (cycle threshold). Ct values were normalized to β-actin, then to the average normalized values of the controls. Primers are listed in Supplemental Table 11.

### RNA-seq, library prep, and analysis.

Libraries were prepared with the NEBNext poly(A) Magnetic Isolation Module (New England Biolabs [NEB], E7940L) followed by the NEBNext Ultra Directional RNA library preparation kit (NEB E7420L/E7760L). Libraries were assessed with an Agilent BioAnalyzer 2100 and quantified with the Library Quant Kit for Illumina (NEB E7630L). Libraries were sequenced with the Illumina NextSeq 500 using 75 bp single-end reads. Reads were mapped to *Mus musculus* UCSC mouse GRCm38/mm10 reference genome with RNA STAR aligner (v2.6.1a) (52). Transcripts per million generation and differential expression were performed using DESeq2 (v1.0.1) (53). Statistical significance was obtained using adjusted *P* value (padj) < 0.05. Log_2_fold-change cutoffs are as indicated. GO analyses were performed using PANTHER (54) under “biological process” with either FDR or Bonferroni correction. The plotted terms represent the highest fold enrichment under PANTHER’s hierarchical clustering. GO and Kyoto Encyclopedia of Genes and Genomes pathway analyses were also conducted using g:Profiler (55). Plotted terms represent top driver terms as by g:Profiler’s “highlight drivers” function.

### ChIP-seq and analysis.

ChIP-seq was performed as described previously (25). Keratinocytes were fixed in 1% formaldehyde for 5 minutes and quenched with 125 mM glycine. Cells were scraped, washed, and sonicated for 15 minutes. All ChIPs were performed using 500 μg of extract, 2 μg of antibody per sample, and 30 μL of Protein G Dynabeads (Thermo Fisher Scientific Fisher 10004D) per ChIP. Library prep was with the NEBNext Ultra DNA library preparation kit (NEB E7645L). Quality check and sequencing were performed as above. ChIP-seq reads were aligned to human reference genome (hg19) using bowtie2 (v2.1.0) (56) and filtered for unique reads. Aligned reads from biological replicates were pooled. HOMER v4.10.1 (57) was used to generate tag directories for pooled samples; call peaks (using corresponding inputs and default parameters); and generate tracks (allowing a maximum of 1 tag per base pair to remove PCR duplicates, normalizing experiments to 10 million total tags, and subtracting corresponding input (-tbp 1, -i -subtract). Tracks were visualized using UCSC Genome Browser (58).

### Murine single-cell isolation for CUT&RUN.

Mouse dorsal skin was shaved, and treated with Nair (Church & Dwight) to completely remove all remaining hair, and underlying fat pad removed by scraping. Skin was floated dermis-side down on 0.25% trypsin (Gibco 15090-046) for 45 minutes at 37°C. The epidermis was scraped into DMEM (Gibco 11965-084) with 10% FBS (Hyclone SH30910.03). For H3K4me2 CUT&RUN, epidermis was broken with scissors, incubated with Liberase (Roche, 05401119001) in 1× HBSS with Ca^2+^/Mg^2+^ (Gibco 14025-092) and 42 mM HEPES (Gibco 15630-080) supplemented with DNase I (50 U/mL, Roche 04716728001) at 37°C for 60 minutes at 68*g*, and quenched with 3 μL of 0.5 M EDTA (Gibco 15575-038) and FBS. The suspension was filtered through a 70 μM cell strainer (Thermo Fisher Scientific 22363548). The solution was centrifuged for 5 minutes at 1188*g*, resuspended in 1 mL of PBS without Ca^2+^ and Mg^2+^ (Gibco, 14190-36) + 1% BSA (Sigma, A7906-100g), and centrifuged again. The pellet was resuspended in 1 mL of 0.04% BSA in PBS without Ca^2+^ and Mg^2+^ (Gibco, 14190-36) for counting. For LSD1 CUT&RUN epidermis was dissociated by pipetting and suspension filtered through 100 μM cell strainer (Thermo Fisher Scientific 22363549) then 40 μM cell strainer (Thermo Fisher Scientific 22363547). Cells were centrifuged for 10 minutes at 300 rcf, and the pellet was resuspended in 1 mL of 50/50 NHEK media. All cells were counted using an automated cell counter (Invitrogen, Countess).

### CUT&RUN.

H3K4me2 CUT&RUN was performed using the CUT&RUN Assay Kit (Cell Signaling Technology, 86652S). A total of 200,000 cells from either WT or LSD1-KO mice were used. Antibodies included H3K4me2 (Abcam ab7766, 2 μL), H3K4me3 (Cell Signaling Technology 9751T, 2 μL), and IgG (Cell Signaling Technology 66362S, 5 μL) (Supplemental Table 12). DNA was extracted with spin columns (Cell Signaling Technology 142095S). LSD1 CUT&RUN followed Henikoff lab protocols (59) and CUT&RUN Assay Kit. Antibodies included LSD1 (Abcam, ab1772, 1:50 dilution) and IgG (Cell Signaling Technology 66362S, 1:20 dilution, negative control) (Supplemental Table 12). A total of 350,000 cells from WT mice were cross-linked with formaldehyde (Fisher Chemical, F79-500), rotated for 2 minutes at room temperature (RT), and quenched with Glycine Solution (7005S, Cell Signaling Technology). Reverse cross-linking was performed at 65°C for 2 hours. DNA was extracted using phenol/chloroform (Fisher Bioreagents, BP 17521-400) with Phase Lock Gel Heavy (Quantabio, 2302830). Ethanol precipitation was carried out overnight at –20°C, and the pellet was resuspended in 17 μL of nuclease-free water following centrifugation. DNA concentration was measured by Qubit dsDNA HS Assay kit (Life Technologies Q32851). NEBNext Ultra DNA library prep kit for Illumina (NEB E7645S) was used for library preparation. Size selection was performed using AMPure XP Beads (Beckman Coulter A63881), for H3K4me2 samples, 0.4× followed by 1×, and for LSD1 samples, 0.8× followed by 1.2×. Libraries were assessed on BioAnalyzer 2100 and quantified with the Library Quant Kit for Illumina. Libraries were denatured, diluted to 1.8 pM, and sequenced using 37-37 bp paired-end reads.

### CUT&RUN data analysis.

Reads were aligned to mm10 mouse and *Saccharomyces cerevisiae* (SacCer3) genomes using bowtie2 (v2.3.4.3) (56) with parameters --local --very-sensitive --no-unal --no-mixed --no-discordant --phred33 -I 10 -X 700 --dovetail. After alignment, files were converted to BAM, then filtered to remove unmapped reads and (-F 4) reads with a mapping quality < 10 (-q 10) using samtools v1.9 (60). BAM alignment files were sorted and filtered to return reads mapped in proper pair (samtools view-bf 0x2) and converted to paired-end BED (bedtools bamtobed with-bedpe option) (v2.27.1) (61). BED files were sorted by position, with both 5′ and 3′ read pair coordinates extracted, then converted to bedGraph via bedtools, with regions without signal left out. Spike-in normalization was applied through a normalization factor (lowest number of unique reads aligned to spike-in genome reference divided by number of unique reads aligned to spike-in genome reference in each sample (genomecov-bg-scale). H3K4me2 peak identification on normalized bedGraph files was with SICER2 peak caller (v1.0.2) (62). Differential H3K4me2 peaks were pinpointed using the sicer_df function with resolution window size 200 bp (--w 200) and minimum length of “gap” such that the neighboring window was an “island” sized to be 600 bp (--f 600). Significant differential H3K4me2 regions were defined as padj or FDR < 0.01. For LSD1 CUT&RUN data, raw reads were checked by FastQC. Paired-end reads were aligned to the mm10 reference genome using bowtie2 (v2.1.0) (56) with parameters -q --local --very-sensitive --no-mixed -p 10 --no-unal --phred33. Resulting BAM files were filtered using samtools to keep reads with mapping quality score greater than 20 (-q 20) (60). Duplicate reads and blacklist regions were further removed using picard MarkDuplicates (v2.23.4) (63) and bedtools intersect (v2.30.0) (61), respectively. The filtered files were converted into BigWig for visualization using deeptools (v3.5.4) (64) bamCoverage with parameters: --binSize 10 --normalizeUsing RPKM. LSD1 peaks were called over IgG control using macs2 (65) with parameters: -f BAM -g mm -q 0.05 --keep-dup all. Peak annotation was with ChIPseeker (v1.44.0) (66) and GREAT (v4.0.4) (67). Enrichment was visualized with UCSC Genome Browser (58). HOMER de novo and known motif finding was performed through findMotifsGenome.pl with custom -bg parameter from an epithelial gene set (57). GO analyses were as above.

### T cell depletion studies.

In homeostatic models, 100 μg of anti-CD4 (Clone GK1.5, InVivo MAb BE0003-1) and 100 μg of anti-CD8 (Clone 2.43, InVivo MAb BE0061) in PBS was injected by i.p. on day –2, 0, 5, 7, and 9 of topical treatment (first day as day 0) (68). As control 200 μg IgG (Clone LTF-2, InVivo MAb BE0090) in PBS was injected i.p. For the DMBA-TPA model, 100 μg of anti-CD4 (Clone GK1.5, InVivo MAb BE0003-1) in PBS or 100 μg IgG (Clone LTF-2, InVivo MAb BE0090) in PBS was injected by i.p. on day –7, –5, 0, 2, 4, 9 of topical. Mice were harvested on day 15. Spleen was taken for flow to validate depletion.

### Flow cytometry.

For skin, epidermis was taken for single-cell suspension as above and filtered through a 70 μm cell strainer (Thermo Fisher Scientific 22363548). Lymph nodes were passed through a 70 μm filter. Samples were treated with RBC Lysis Buffer (Tonbo Biosciences), washed in FACS buffer (1× PBS, 2% FBS, 2 mM EDTA, 25 mM HEPES), and Fc receptor–blocked with CD16/32 (2.4G2, 1:50, BD). Cells were stained with anti-mouse fluorochrome-conjugated monoclonal antibodies (Supplemental Table S13). For IL17 staining, cells were stimulated with 50 ng/mL of PMA and 1 mg/mL of ionomycin for 3 hours. Protein transport inhibitor (Thermo Fisher Scientific, 4980) was added for the last 2 hours. All cells were fixed and permeabilized using the eBioscience Intracellular Fixation & Permeabilization Buffer Set (Invitrogen, 88-8824-00). Flow data were collected with an Aurora flow cytometer (Cytek) and analyzed using FlowJo (BD).

Spleens were passed through a 70 μm cell strainer (Miltenyi Biotec 130-110-916). Tumors were dissociated with scissors, incubated in collagenase + DNase I at 37°C for 45 minutes, and centrifuged at 500*g* for 5 minutes at 4°C. For spleens and tumors, pellets were treated with ACK Lysis Buffer (Thermo Fisher Scientific A1049201) for 5 minutes, then washed with FACS buffer (1× PBS, 2% FBS, 1 mM EDTA). Samples were stained with viability dye in PBS for 15 minutes at 4°C, then with cell surface marker antibodies for 30 minutes at 4°C in FACS buffer. Cells were washed with FACS buffer, fixed in 2% paraformaldehyde for 10 minutes at 4°C, and resuspended in FACS buffer. Data were collected using an LSR II (BD) and analyzed using FlowJo.

Antibodies used for flow are listed in Supplemental Table 13. Panels 1 and 2 were used for live and fixed flow of skin, respectively. Panel 3 was used for fixed flow of skin dLNs. Panel 4 was used for spleens. Panel 5 was used for tumors.

### Immunoblotting.

Lysates were quantified with Quick Start Bradford 1× Dye Reagent (Bio-Rad 50000205) with 20–30 μg used. Lysates were boiled, loaded on a 4%–20% SDS-polyacrylamide gel, and transferred to a PVDF membrane for 1 hour at 100 V at 4°C. Membrane was blocked with 5% milk at RT for 1 hour and blotted with primary antibodies (Supplemental Table 12) overnight at 4°C. Secondary antibodies at 1:5,000 (Cell Signaling Anti-Rabbit IgG 7074 and Anti-Mouse IgG 7076) and ECL Prime Western Blotting Detection Reagents (Amersham RPN 2232) were used. Membranes were imaged on a ChemiDoc Imaging system (Bio-Rad).

### IHC and IF.

Slides were baked for 1 hour at 65°C, deparaffinized with 2× 7-minute xylene washes, and rehydrated with ethanol gradient. Slides were treated with antigen-unmasking solution (citric acid 1:100, Vector Laboratories H-3300) for 15 minutes at 95°C, hydrogen peroxide for 14 minutes at RT, and 0.5% Triton-X in TBS-Tween (TBST) for 5 minutes. Protein block was applied for 1 hour, and then primary antibodies (Supplemental Table 12) were used overnight at 4°C. After TBST washes, secondary antibody was applied for 20 minutes at RT followed by DAB stain with exposure times synchronized. Hydrogen peroxide, blocking, secondary antibody, and DAB were done using reagents from the Mouse and Rabbit Specific HRP/DAB IHC Detection Kit (Abcam 236466). Slides were counterstained with hematoxylin for 10 seconds followed by dehydration with an ethanol-xylene gradient. Slides were mounted with VectaMount (Vector Laboratories H-5000). For immunofluorescence, after antigen retrieval, slides were treated with 0.5% Triton-X in TBST for 5 minutes. Protein block was applied for 1 hour, and primary antibodies (Supplemental Table 12) were used overnight at 4°C. Slides were washed with 1× TBST followed by secondary antibody diluted 1:500 for 1 hour. Secondary antibodies included Alexa Fluor 488 Anti-Rabbit IgG (Invitrogen A21206), Alexa Fluor 555 Anti-Mouse IgG (Invitrogen A31570), and Alexa Fluor 488 Anti-Goat IgG (Invitrogen A32814). Slides were mounted with ProLong Gold antifade reagent with DAPI (Invitrogen P36935). All slides were imaged with a Leica DM6B microscope.

### Xenium in situ spatial transcriptomics.

FFPE blocks were sectioned at 5 μm thickness onto Xenium slides. According to the 10x Genomics Xenium protocol (Protocol: CG000580, CG000582, CG000584), slides were baked for 2 hours at 60°C followed by deparaffinization in xylene and rehydration with ethanol gradient. Slides were de-cross-linked: 80°C for 30 minutes, 22°C for 10 minutes. mRNAs were targeted with a custom 480-gene panel. The 480 genes were based on pathways of interest from RNA-seq in the selected tissues and from cell type markers in the 10x Genomics Human Skin Panel. Genes were checked against a reference single-cell RNA-seq dataset (*Tabula Muris Senis*) to determine lowly or highly expressed genes (69), and probes were adjusted accordingly. Probes were resuspended in TE buffer and preheated by incubating for 2 minutes at 95°C. Hybridization was performed overnight (16–24 hours) at 50°C. The following day, posthybridization wash was performed at 37°C for 30 minutes. Probes were ligated at 37°C for 2 hours and amplified at 30°C for 2 hours with PBST washes in between. Slides were stained with reducing agent (10 minutes), then autofluorescence quenching (10 minutes), then ethanol washes and drying at 37°C for 5 minutes. Nuclei were stained with DAPI for 1 minute. Xenium imaging processing, decoding, quality score generation, and DAPI-based segmentation were performed by the Xenium Analyzer system as described (70).

### After Xenium H&E staining.

After removal from the Xenium analyzer, slides were washed with PBST, incubated in 0.88 g of sodium hydrosulfate in 50 mL MilliQ H_2_O (MilliporeSigma) for 10 minutes at RT, washed 3 times for 1 minute each in MilliQ H_2_O, and incubated in hematoxylin for 3.5 minutes (Surgipath 3801540). Slides were washed with tap water 5 times for 1 minute each, then destained in 1% acid alcohol (10 mL HCl in 1,000 mL 70% EtOH) followed by tap water. Slides were incubated in bluing solution for 1.5 minutes (Azer Bluing Solution E5745-1G), washed in tap water 3 times for 1 minute each, dipped in 95% EtOH twice, and incubated in eosin for 1 minute (Surgipath 3801600). Last, they were dehydrated in an ethanol gradient, cleared in xylene substitute (Everclear, Azer E5657), and mounted with mounting media (Surgipath 3801120). H&E sections were imaged with a slide scanner, converted to.ome.tiff file using QuPath (v0.5.1), and aligned using Xenium Explorer.

### Xenium data analysis.

Files were loaded into Seurat (v4.3.0 or 5.0.3) with ReadXenium. Fields of view were loaded using centroids and nuclei segmentation data. For CreERT LSD1-KO mice, Seurat objects were merged maintaining original identities. For Xenium on embryos or tumor-bearing mice, objects were separate. Cells with 0 counts were filtered out. SCTransform normalization was performed, then RunPCA (npcs = 30), then FindNeighbors (dims = 1:30). Final clustering was performed with the Louvain algorithm, Modularity optimizer (v1.3.0), with resolution set to 0.4. FindAllMarkers was run on clusters to find top genes for manual cell type annotation using accompanying histology and the 10x Genomics Xenium Human Skin annotations and skin single-cell RNA-seq databases as guides (71, 72). Visualization was performed with DimPlot, DotPlot, and FeaturePlot in Seurat. Spatial images and aligned H&E images were taken from Xenium Explorer.

For differential gene expression, a Wilcoxon rank-sum test using FindAllMarkers was performed between annotated clusters. Alternatively, cell types were subdivided based on gene expression (i.e., embryonic basal keratinocytes defined as *Krt5*^+^*Col17a1*^+^ cells; T cells defined as *Cd3d*-, *Cd3g*-, or *Cd3e*-expressing cells). Differential expression between 2 conditions for a cell type was done using DESeq2 and compared against results from the Wilcoxon test. GO analyses were as above.

For retinoid and immune gene coexpressing cell identification, regions of interest (ROIs) were selected using Xenium Explorer to obtain sample-specific spatial data. These data were divided to contain cells expressing any one of the following immune genes (*Ccr7*, *Ccl20*). The cells were divided again to filter out cells expressing any one of the following retinoid signaling genes (*Aldh1a3*, *Crabp2*) to obtain a population of cells that only expressed immune genes. This “solo” population was subtracted from the total population to obtain the coexpressing cell population. The number of coexpressing cells was divided by total number of cells to calculate the probability of a cell coexpressing retinoid and immune genes. The probability/proportions of co-occurring cells were calculated from each ROI.

### Statistics.

Data analyses were conducted using either R (v4.1.1 or 4.3.3) or GraphPad Prism (v10.2.2). A *P* < 0.05 was considered statistically significant. Specific tests are listed in figure legends. One-way ANOVAs were corrected for multiple comparisons. All experiments contain at least 3 biological replicates per condition.

### Study approval.

Animal experiments were conducted in accordance with and approved by the Institutional Animal Care and Use Committee of the University of Pennsylvania (Protocol 806119). Human donors were obtained deidentified from the Penn SBDRC.

### Data availability.

All data are available in the main text, supplement, and Supporting Data Values (Supplemental Table 14). Sequencing and Xenium data are on NCBI GEO GSE302760.

## Author contributions

NK, KEW, and BCC conceived of the study, designed experiments, and wrote the manuscript. NK performed most experiments, analyzed data, validated results, and wrote the manuscript. AFM performed cell culture experiments and assisted with CUT&RUN. CAD, CO, and LB assisted with mice. SH assisted with ChIP-seq, CUT&RUN, and Xenium analysis. JC, LVP, and AJ performed flow cytometry. SE and DBC assisted with embryos. ASA assisted in murine experiments. JTS performed dermatopathological analyses of murine tissues. CTM and SMP assisted with histology. EKK and SZ assisted with CUT&RUN. YA assisted with ChIP-seq analysis. BCC, KEW, and DAH provided funding and supervision. All authors assisted with review of the final manuscript.

## Conflict of interest

The authors have declared that no conflict of interest exists.

## Funding support

This work is the result of NIH funding, in whole or in part, and is subject to the NIH Public Access Policy. Through acceptance of this federal funding, the NIH has been given a right to make the work publicly available in PubMed Central.

- NIH grants K08AR070289 and R01AR077615 (to BCC).
- Damon Runyon Cancer Research Foundation Clinical Investigator Award (to BCC).
- Dermatology Foundation Stiefel Award for Skin Cancer (to BCC).
- Skin Cancer Foundation Todd Nagel Memorial Award (to BCC).
- NIH grants T32GM007170 and T32AR007465 (to NK).
- NIH grant R01HL162715 (to DAH).
- NIH diversity supplement to R01CA262055 (to LVP and KEW).
- NIH/NIAMS grant P30AR069589 (principal investigator: Elizabeth A. Grice) (to Penn SBDRC).

## Supplementary Material

Supplemental data

Unedited blot and gel images

Supporting data values

## Version 1 03/12/2026

In-Press Preview

## Version 2 04/15/2026

Electronic publication
