# Supplementary material for: Inhibiting LSD1 unlocks retinoid AP-1 programming to activate epithelial immunity and skin tumor suppression
Source: J Clin Invest. 2026 Mar 12;136(8):e189044. doi: 10.1172/JCI189044 (PMC13078892; doi:10.1172/JCI189044)
Supplement: Supplemental data [file jci-136-189044-s139.pdf]

# Supplementary Materials

## Inhibiting LSD1 unlocks retinoid AP-1 programming to activate epithelial immunity and skin tumor suppression

**Authors:** Nina Kuprasertkul[1,2], Alyssa F. Moore[1,2], Carina A. D'souza[1], Julia Chini[4,5], Eun-Kyung Ko[1,2], Sijia Huang[3], Shuo Zhang[2], Ashley S. Anderson[1,2], Shaun Egolf[1,2], Laura V. Pinheiro[6,7], Alison Jaccard[6,7], Claudia T. Magahis[1], Lydia Bao[1], Yann Aubert[1,2], Cyria Olingou[1], Stephen M. Prouty[1], Donna Brennan-Crispi[1], David A. Hill[4,5], John T. Seykora[1,7], Kathryn E. Wellen[2,6,7], Brian C. Capell[1,2,6,7,8]*

Corresponding author: capellb@pennmedicine.upenn.edu

**The PDF file includes:**

Figs. S1 to S12
Tables S11 to S13

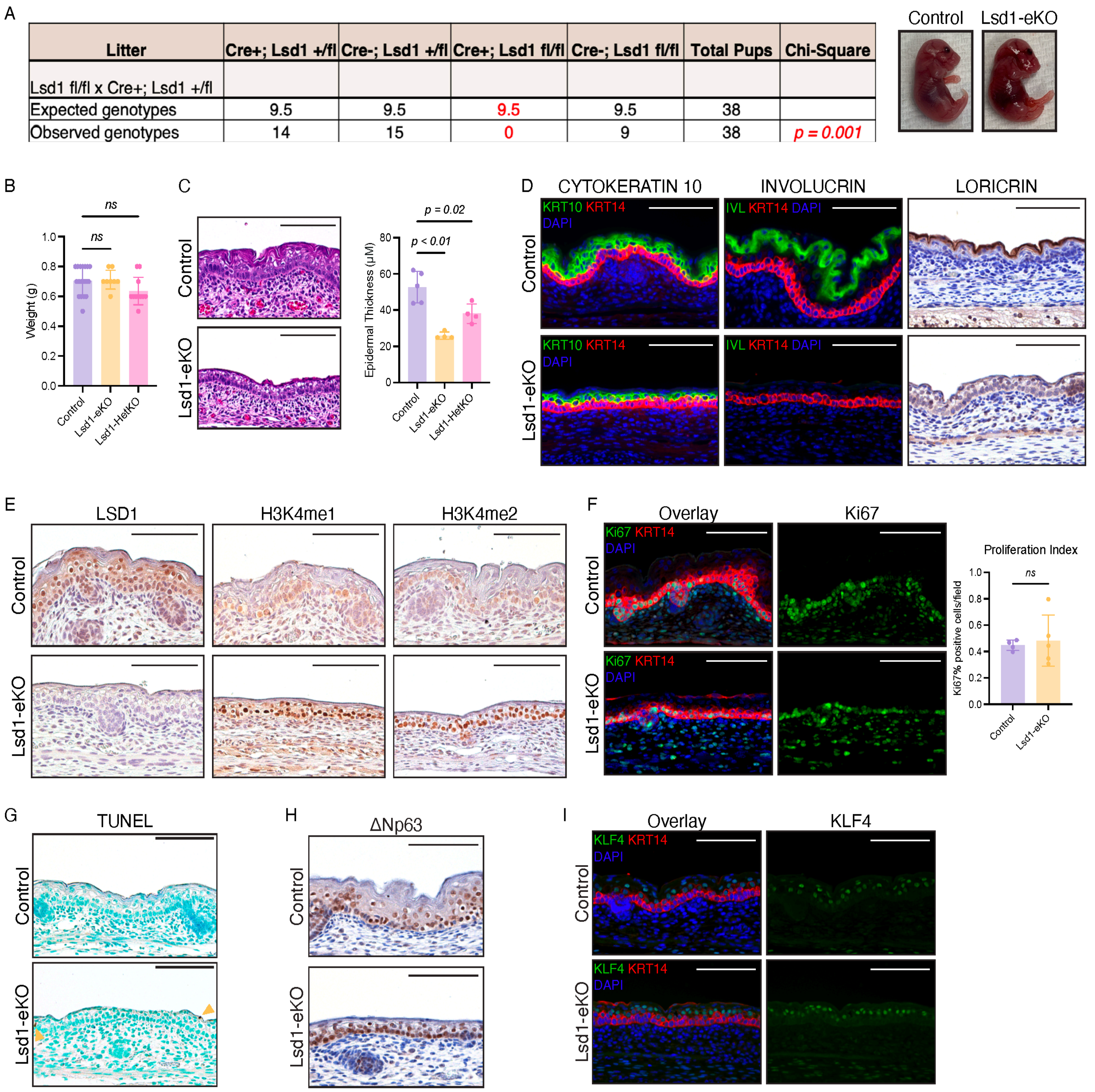


**Fig. S1. LSD1 is required for embryonic epidermal development.** (**A**) Chi-square analysis of pups born from breeding schemes. Genotyping at 3 wks of age (left). Gross skin phenotype from Control and Lsd1-eKO embryos at E17.5 (right). (**B**) Average embryo weights; One-way ANOVA. (**C**) H&E of Control and Lsd1-eKO mice with quantification; One-way ANOVA. (**D**) IF for KRT10 (green) (left); IF for IVL (green) (right); IHC for LOR (right). KRT14 (red) and DAPI (blue). (**E**) IHC for LSD1, H3K4me1, H3K4me2. (**F**) IF for Ki67 (green) with quantification. KRT14 (red) and DAPI (blue). Student's t-test. (**G**) TUNEL staining. (**H**) IHC for ΔNp63. (**I**) IF for KLF4 (green). KRT14 (red) and DAPI (blue). All scale bars: 100μM. Data represented as mean ± SD. Each dot represents an individual embryo: for (A), images representative of 4 independent experiments; for (B), n = 8-18 embryos per genotype; for (C) and (F), n = 4-5 embryos per genotype; for (D) n = 3-6 embryos per genotype; for (E) and (G) to (I), n = 3 representative embryos per genotype for staining. ns = non-significant (p > 0.05)

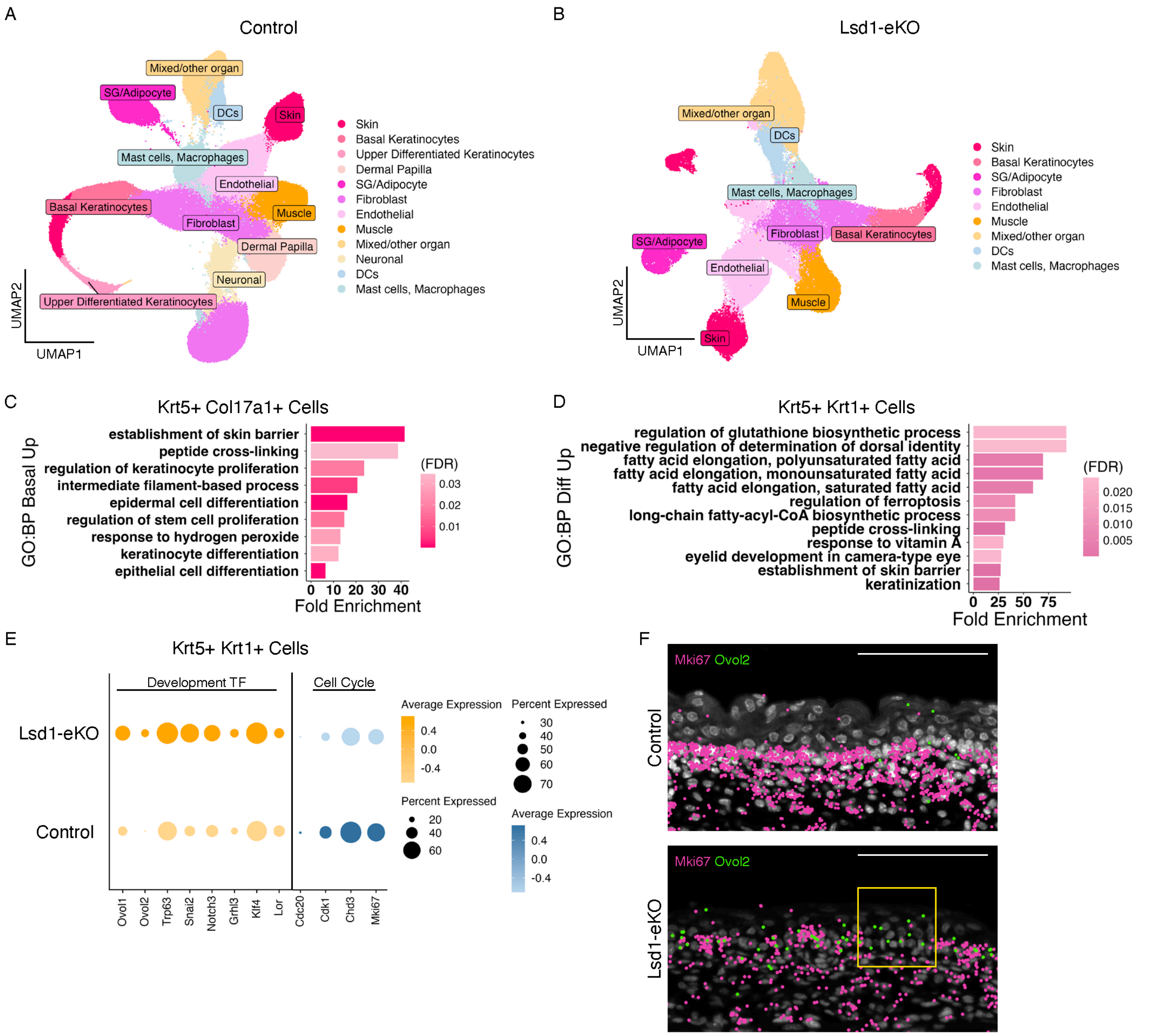


**Fig. S2. LSD1 loss disrupts differentiation programming in embryonic basal and transitional cells.** (**A**) UMAP of annotated clusters from Control Xenium data from E17.5 whole mount and whole skin sections. (**B**) UMAP of annotated clusters from Lsd1-eKO Xenium data from E17.5 sections as in (A). (**C**) GO analysis of upregulated genes from differentiation expression analysis of epidermal basal layer region of interest (ROI) (defined as *Krt5*+, *Col17a1*+) in Lsd1-eKO and Control Xenium sections. (**D**) GO analysis of upregulated genes from differentiation expression analysis of transitional epidermal layer region of interest (ROI) (defined as *Krt5*+, *Krt1*+) in Lsd1-eKO and Control Xenium sections. (**E**) Dotplot of key developmental transcription factors (TFs) and cell cycle genes in Control and Lsd1-eKO Xenium sections. (**F**) Xenium sections in Control and Lsd1-eKO mice with proliferation (*Ki67*) and differentiation (*Ovol2*) genes labeled. Yellow box denotes Ovol2+ transcript dense area with Ki67+ transcript paucity. All scale bars: 100μM. For (A) to (F); n = 4 embryos per genotype. FDR = false discovery rate.

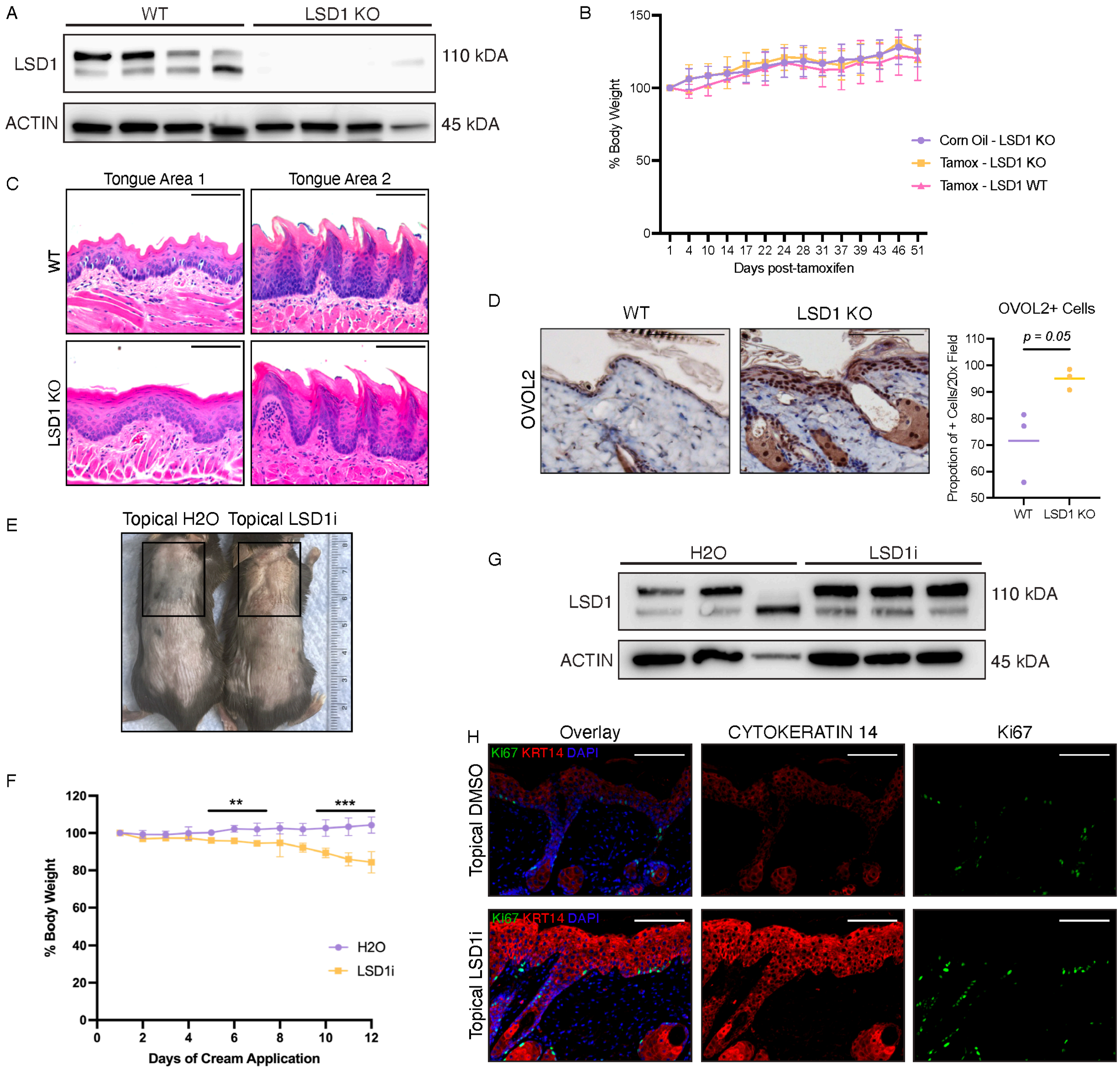


**Fig. S3. Pharmacologic or genetic inhibition of LSD1 disrupts adult skin homeostasis.** (**A**) Western for LSD1 and beta-ACTIN from epidermal lysates of WT and LSD1 KO mice taken at endpoint. (**B**) Weights of WT (vehicle and genetic controls) and LSD1 KO mice normalized to body weight on D1. (**C**) H&E of tongue tissue from WT and LSD1 KO mice (**D**) IHC for OVOL2 in WT and LSD1 KO mice (left). Proportion of positively staining cells per 20x field; Student's t-test (right). (**E**) Gross phenotype of mice treated with either topical H2O or topical LSD1i for 12 days. Image taken post-euthanasia, ruler in cm, box depicts treatment area (**F**) Weights of H2O and LSD1i treated mice normalized to body weight on D1; Multiple unpaired t-tests. (**F**) IF for Ki67 (green). KRT14 (red) and DAPI (blue). All scale bars: 100µM. Data represented as mean ± SD. Each dot represents an individual mouse for bar graphs: for (A), n = 4 mice per condition; for (B) and (F), n = 4-5 mice per condition; for (C), (D), and (H), n = 3 mice per condition for staining; for (E), images representative of 5-6 independent experiments. *p<0.05, **p<0.005, ***p<0.0005.

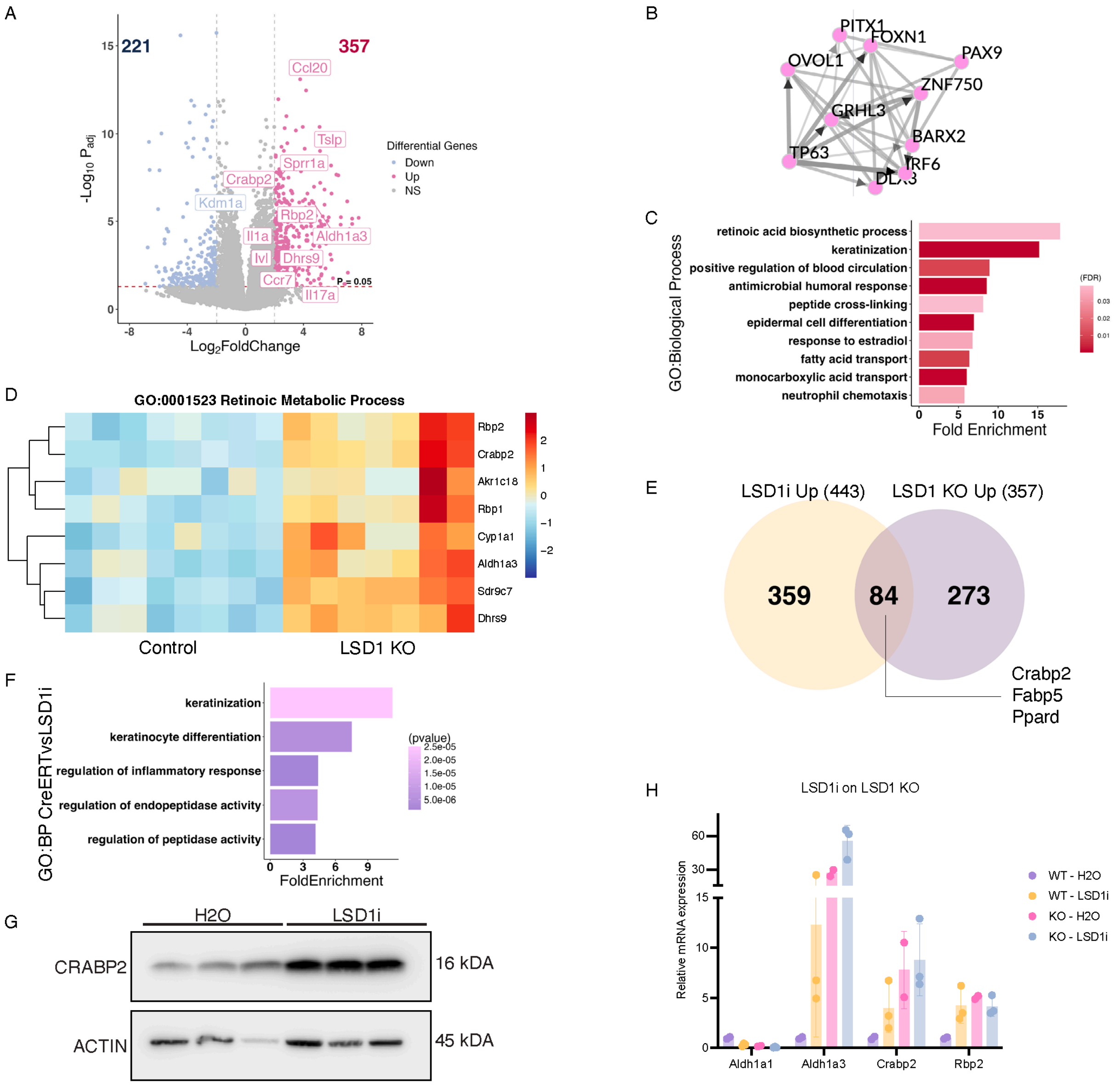


**Fig. S4. LSD1 loss upregulates retinoic acid signaling markers.** (**A**) Volcano plot from RNA-seq of WT and LSD1 KO mice; relevant RA, differentiation, and immune genes highlighted (**B**) ChEA3 TF network analysis of RNA-seq. (**C**) GO analysis of upregulated genes in KO vs WT. GO terms include retinoic acid biosynthetic process (GO:0002138), keratinization (GO:0031424), antimicrobial humoral response (GO:0019730). (**D**) Heatmap with enriched terms from GO:0001523. (**E**) Overlap between upregulated genes in LSD1 KO vs WT mice and topical LSD1i vs topical DMSO mice. (**F**) GO analysis of overlapping upregulated genes. GO terms include keratinocyte differentiation (GO:0030216) and regulation of inflammatory response (GO:0050727). (**G**) Western for CRABP2 and beta-ACTIN in H2O and LSD1i treated mice for 15 days. (**H**) qPCR for retinoid genes in WT and LSD1 KO mice treated with H2O or LSD1i for 15 days at end of induction. For (A) to (D), n = 7-8 mice per condition for LSD1 KO RNA-seq; for (E) and (F), n = 4 mice per condition for LSD1i RNA-seq; for (G), n = 3 mice per condition; for (H), n = 2-3 mice per condition. Data represented as mean ± SD. Each dot represents an individual mouse for bar graphs. FDR = false discovery rate. padj = p-adjusted value.

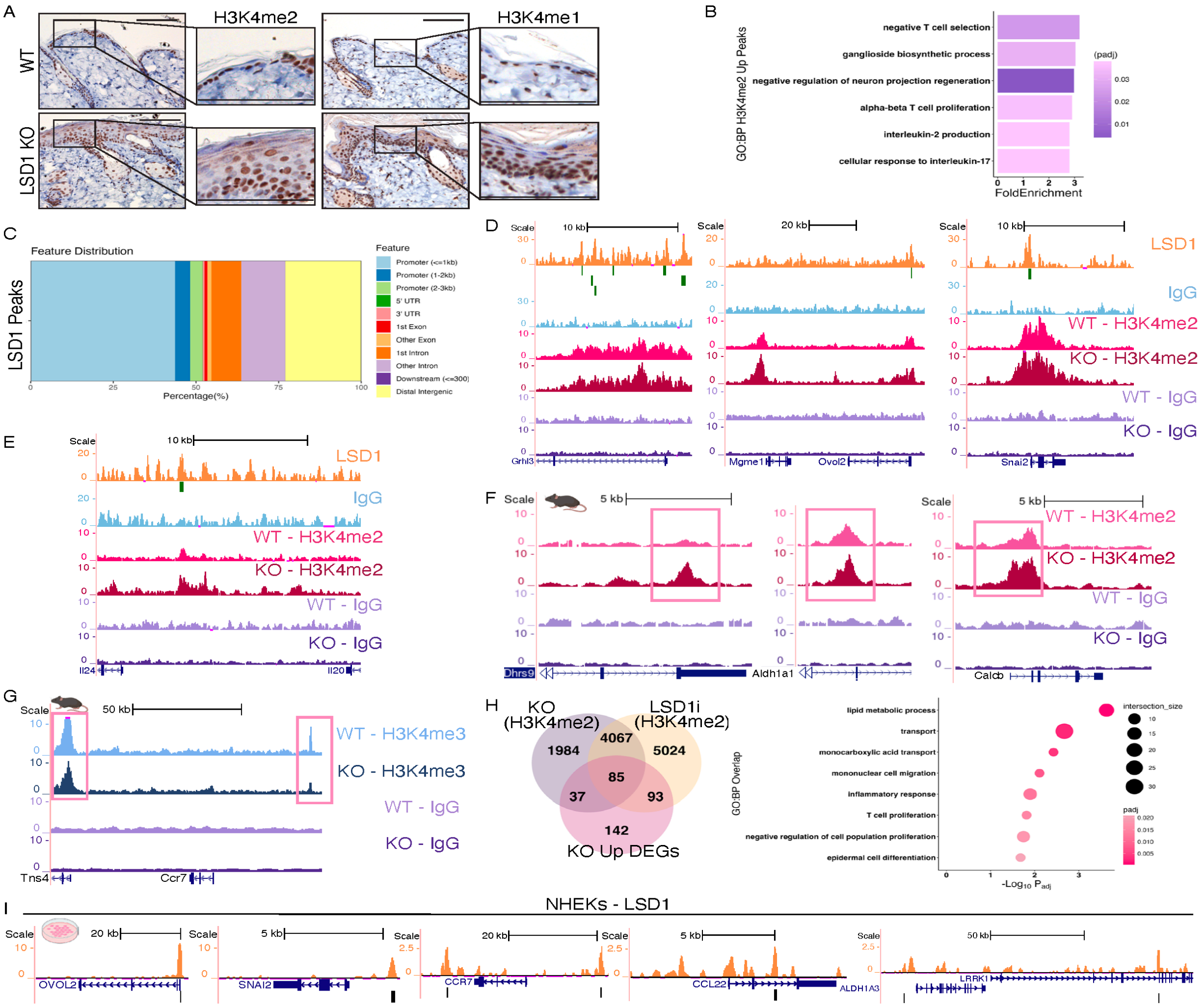


**Fig. S5. LSD1 loss rewires H3K4me2 signals around immune genes.** (**A**) IHC for H3K4me2 (left) and H3K4me1 (right) in WT and LSD1 KO mice. (**B**) GO analysis of genes associated with increased H3K4me2 regions in LSD1 KO vs WT mice. GO terms include alpha-beta T cell proliferation (GO:0046633) and cellular response to interleukin-17 (GO:0097398). (**C**) Peak annotation of LSD1 peaks in WT mice. (**D**) LSD1 tracks in WT mice with H3K4me2 tracks in WT and LSD1 KO mice at *Grhl3, Ovol2, Snai2*. (**E**) LSD1 tracks in WT mice with H3K4me2 tracks in WT and LSD1 KO mice at *Il20, Il24*. (**F**) H3K4me2 tracks in WT and LSD1 KO mice at retinoid metabolism (*Dhrs9*, *Aldh1a1*) and retinoid target (*Calcb*) genes. (**G**) Corresponding H3K4me3 tracks in WT and LSD1 KO mice at *Ccr7* (see Fig.2H). (**H**) Overlap between genes associated with increased H3K4me2 regions in LSD1 KO vs WT mice, upregulated genes in LSD1 KO vs WT mice, and genes associated with increased H3K4me2 regions from LSD1i vs H2O treated mice for 48hrs (left). GO analysis of 85 gene overlap (right). GO terms include lipid metabolic process (GO:0006629), inflammatory response (GO:0006954), and epidermal cell differentiation (GO:0009913). (**I**) LSD1 tracks in proliferating NHEKs at *OVOL2, SNAI2, CCR7, CCL22, ALDH1A3*. For all tracks, mouse icon represents data from CUT&RUN in WT and LSD1 KO mice, culture dish icon represents LSD1 peaks from ChIP-seq in NHEKs. All scale bars: 100µM. For (A), n = 3 mice per condition for staining; for (B) to (H), LSD1 KO CUT&RUN data represents n = 2-3 replicates per condition, pooled from 4-5 mice per condition. LSD1i CUT&RUN data represents n = 3 replicates per condition, pooled from 3 mice per condition (see Methods); for (I), ChIP-seq data represents n = 3 human donors.

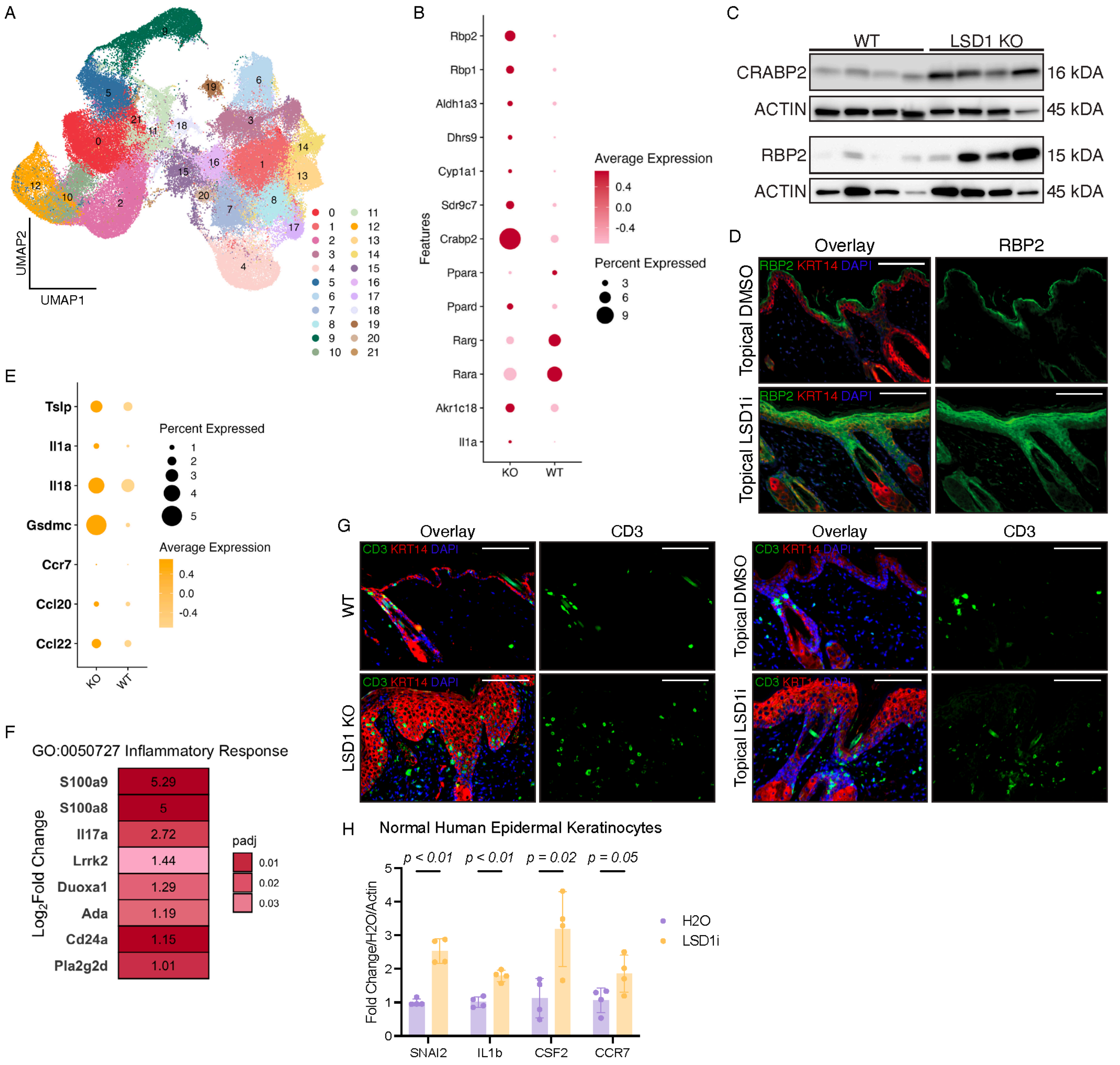


**Fig. S6. LSD1 deficiency increases expression of retinoid and immune genes.** (**A**) UMAP of Seurat clusters from merged WT and LSD1 KO Xenium data. (**B**) Dotplot of RA metabolism genes from Xenium data. (**C**) Western for CRABP2 and RBP2 along with respective beta-ACTIN from epidermal lysates of WT and LSD1 KO mice. Beta-ACTIN blot is identical to Supplementary Figure S3A and was used as the shared loading control for LSD1 and CRABP2. (**D**) IF for RBP2 (green) in topical DMSO or topical LSD1i treated mice. KRT14 (red) and DAPI (blue). (**E**) Dotplot of immune genes from Xenium data. (**F**) Genes in enriched GO term inflammatory response (GO:0050727) from RNA-seq of topical LSD1i vs topical DMSO. (**G**) IF for CD3 (green) in WT or LSD1 KO mice (left) and topical LSD1i or topical DMSO treated mice (right). KRT14 (red) and DAPI (blue). (**H**) qPCR for differentiation and immune genes in NHEKs treated with H2O or LSD1i for 48 hrs; Multiple unpaired t-tests. All scale bars: 100µM. Data represented as mean ± SD. Each dot represents an individual donor for bar graphs: For (A), (B), and (E), n = 4-5 mice per condition; for (C), n = 4 mice per condition; for (D) and (G), n = 3 mice per condition for staining; for (F), n = 4 mice per condition for RNA-seq; for (H), n = 4 human donors per condition.

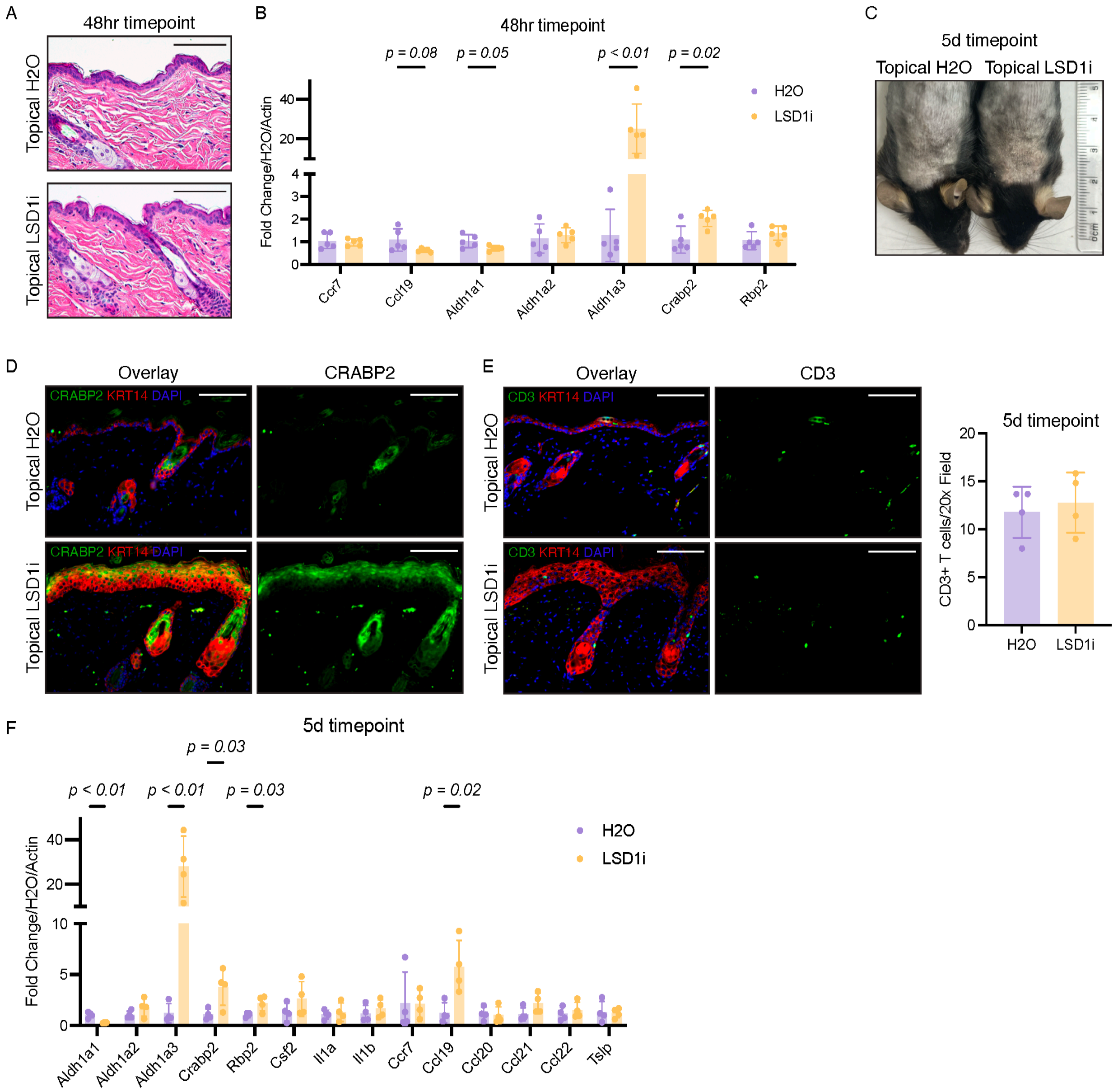


**Fig. S7. LSD1 inhibition leads to early activation of retinoid signaling.** (**A**) H&E of 48-hour topical H2O or LSD1i treated mice. (**B**) qPCR for retinoid metabolism or immune genes in 48-hour treated mice. (**C**) Gross phenotype of skin from 5-day topical H2O or LSD1i treated mice. (**D**) IF for CRABP2 (green) in 5-day treated mice. KRT14 (red) and DAPI (blue). (**E**) IF for CD3 (green) in 5-day treated mice. KRT14 (red) and DAPI (blue). Quantification on right; Student's t-test. (**F**) qPCR for retinoid metabolism or immune-related genes in 5-day treated mice. Multiple unpaired t-tests unless otherwise indicated. All scale bars: 100µM. Data represented as mean ± SD. Each dot represents an individual mouse: for (A) and (B), n = 5 mice per condition; for (C) to (F), n = 4 mice per condition. All significant p-values shown.

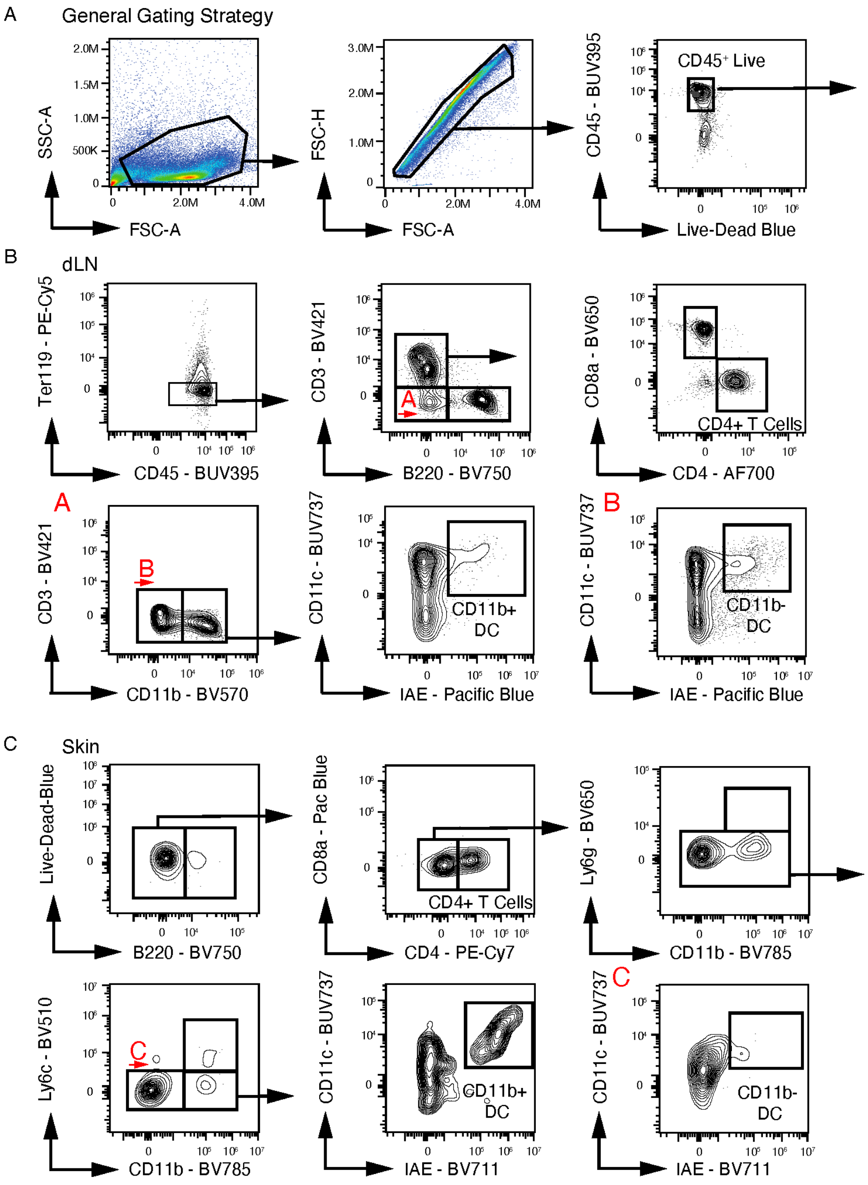


**Fig. S8. Gating strategy for flow cytometry of LSD1i treated mice.** (**A**) General gating strategy used in flow cytometry from topical H2O or LSD1i treated mice for CD45+ live immune cells. (**B**) Gating strategy for CD4+ T cells and dendritic cells (CD11b+ and CD11b-) from draining lymph nodes (dLNs) of topical H2O or LSD1i treated mice. (**C**) Gating strategy for CD4+ T cells and dendritic cells (CD11b+ and CD11b-) from epidermis of topical H2O or LSD1i treated mice. All cells were gated as CD45+ Ter119-. CD4+ T cells, CD11b- DCs, and CD11b+ DCs were gated as shown in (A) to (C). Other cells were gated as follows: CD8+ T cells: B220- CD8+; Eosinophils: B220- CD4- CD8- SiglecF+; Basophils: B220- CD4- CD8- SiglecF- Fcer1a+; Langerhans: B220- CD4- CD8- SiglecF- Ly6g- CD207+ IAE+.

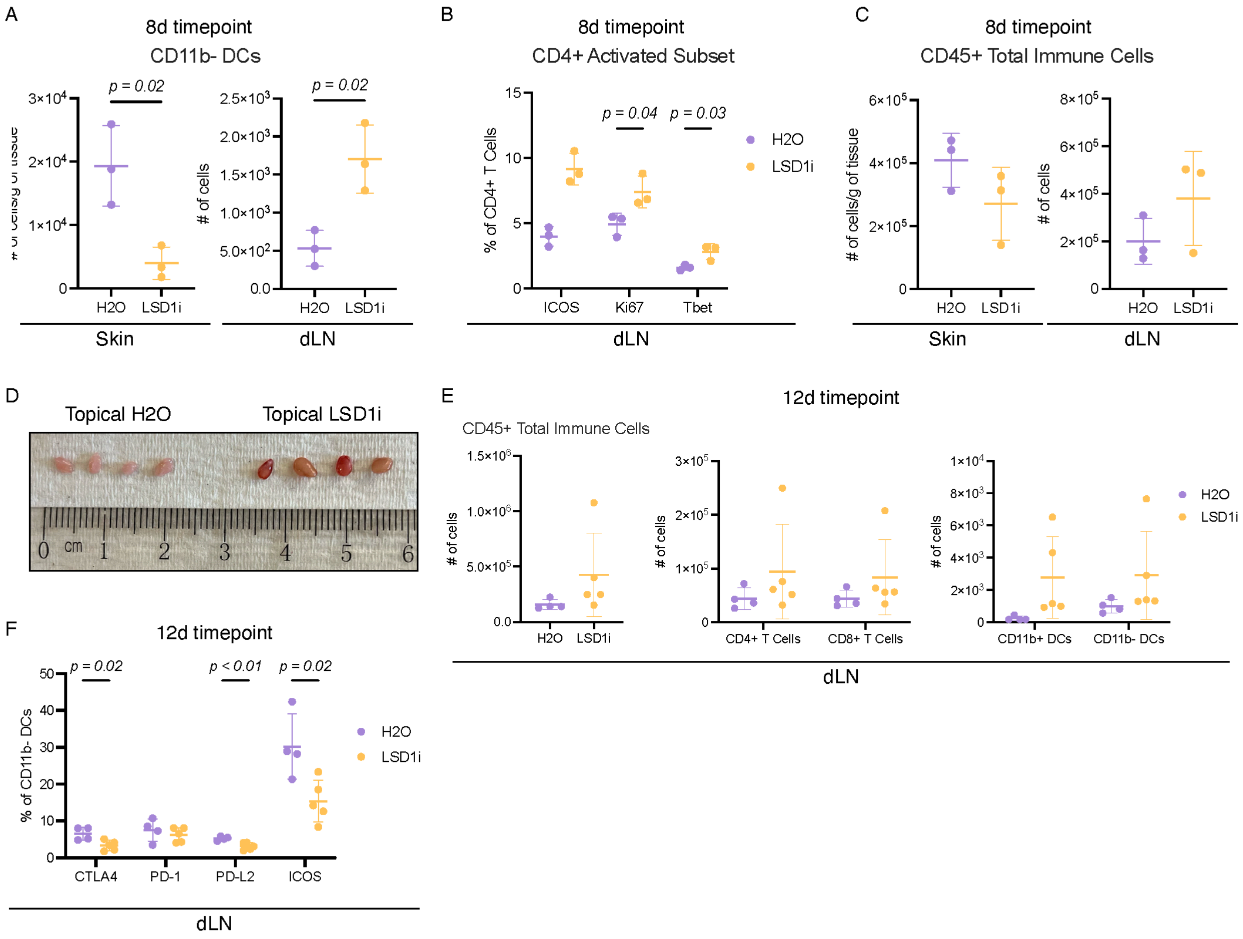


**Fig. S9. LSD1 inhibition leads to early activation of immune cells in dLNs followed by recruitment to the skin.** (**A**) CD11b- DC counts in epidermis or axillary skin dLNs of 8-day topical H2O or LSD1i treated mice. (**B**) Activation markers in CD4+ T cells of 8-day treated mice; Multiple unpaired t-tests. (**C**) CD45+ live cells in epidermis or dLNs of 8-day treated mice. (**D**) dLNs harvested from 12-day topical H2O or LSD1i treated mice. (**E**) Immune cell live counts from dLNs of 12-day treated mice. (**F**) Checkpoint (CTLA4, PD-1, PD-L2) or activation (ICOS) markers in CD11b- DCs from dLNs of 12-day treated mice; Multiple unpaired t-tests. dLN = draining lymph node. Data represented as mean ± SD. Each dot represents an individual mouse: for (A) to (C), n = 3 mice per condition per tissue; for (D), n = 4 mice per condition; for (E) to (F), n = 4-5 mice per condition. Student's t-test unless otherwise indicated.

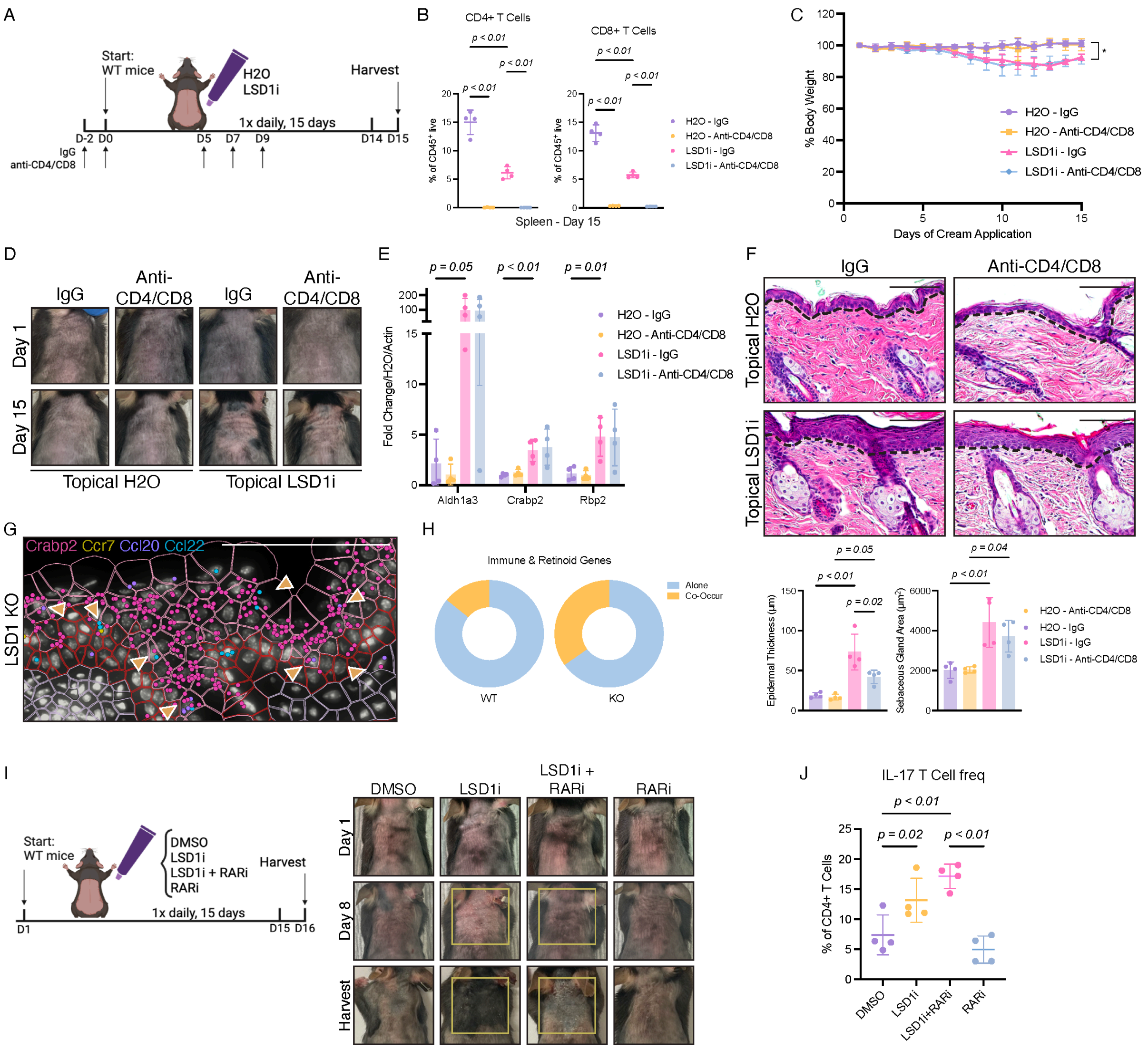

**Fig. S10. LSD1 mediates retinoic acid-dependent immune signaling.** (**A**) T cell depletion schematic. (**B**) Spleen flow cytometry at endpoint for CD4 and CD8 T cell frequency; One-way ANOVA. (**C**) Average weights of topical H2O or LSD1i treated mice with or without T cell depletion; Multiple unpaired t-tests (p-value represents t-test on last day). (**D**) Gross skin phenotypes of T cell depletion cohort on treatment day 15. (**E**) qPCR for retinoid metabolism genes in T cell depleted mouse cohort; Multiple unpaired t-tests. (**F**) H&E of H2O and LSD1i treated mice with IgG or Anti-CD4/CD8 with quantification; One-way ANOVA. Dotted line indicates dermal-epidermal junction. (**G**) Xenium section in LSD1 KO mice (same section as Figure 4D) with additional retinoid metabolism (*Crabp2*) and immune (*Ccr7*, *Ccl20*, *Ccl22*) genes labeled. Yellow arrows point to co-expressing cells. (**H**) Pie chart representation of proportion of retinoid and immune genes being co-expressed upon LSD1 KO. (**I**) LSD1i + RARi rescue study schematic (left). Gross skin phenotypes of DMSO, LSD1i, LSD1i + RARi, and RARi treated mice at treatment days 1, 8, and harvest (right). (**J**) IL17+ CD4+ T cell frequency from skin of LSD1i + RARi cohort mice; One-way ANOVA. All scale bars: 100μM. Data represented as mean ± SD. Each dot represents an individual mouse for bar graphs: for (B) to (F), n = 4 mice per condition; for (H), n = 4-5 mice per condition; for (I) and (J), n = 4 mice per condition. Student's t-test unless otherwise indicated. *p<0.05.

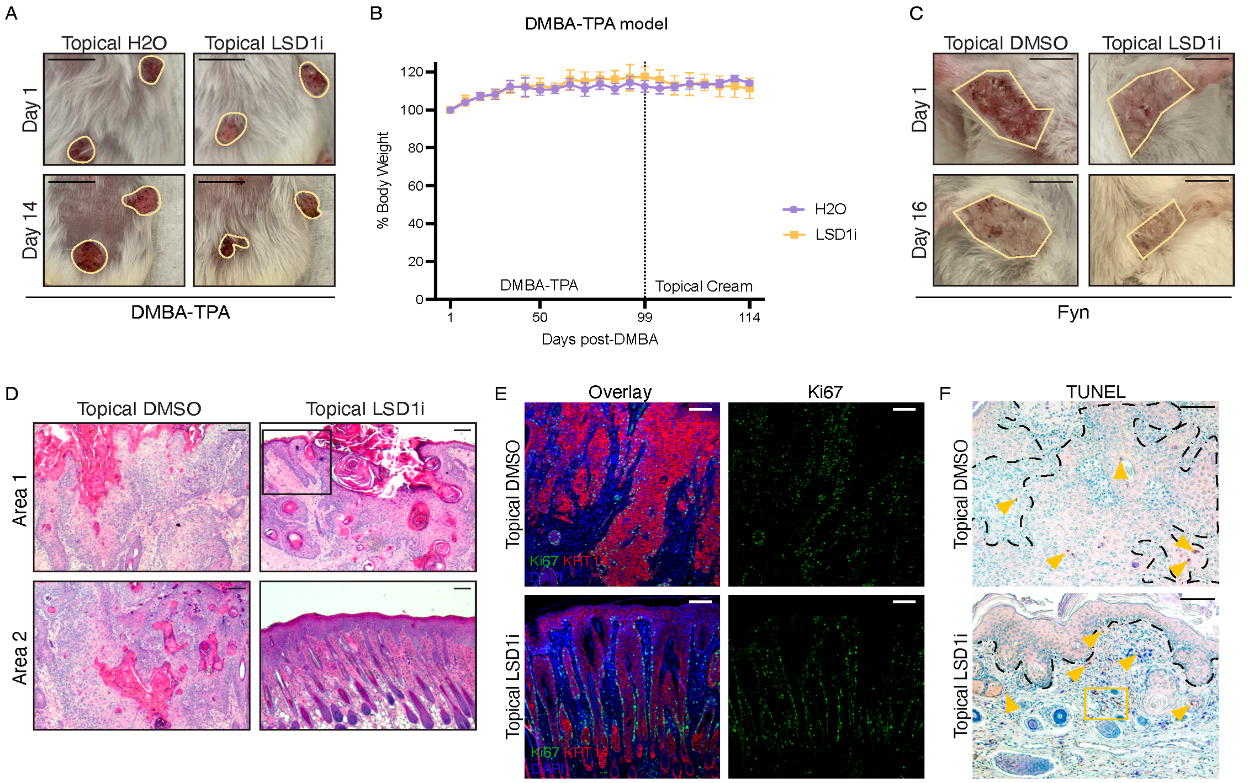


**Fig. S11. LSD1 inhibition is efficacious in two cSCC models.** (**A**) Location matched tumors from DMBA-TPA induction treated with H2O or LSD1i. Scale bar: 1cm. (**B**) Weights of mice throughout DMBA-TPA induction and topical cream treatment. (**C**) Location matched tumors from Fyn mice (genetic induction) treated with DMSO or LSD1i. Scale bar: 1cm. (**D**) H&E of topical LSD1i or DMSO treated tumors from Fyn model taken post-euthanasia. (**E**) IF for Ki67 (green) in Fyn mice treated with topical DMSO or LSD1i. KRT14 (red) and DAPI (blue). (**F**) TUNEL staining in Fyn mice treated with topical DMSO or LSD1i. All scale bars: 100µM unless indicated. Data represented as mean ± SD. For (A) and (B), n = 4-5 mice per condition; for (C) and (D), n = 3-4 mice per condition; for (E) and (F), n = 3 mice per condition for staining.

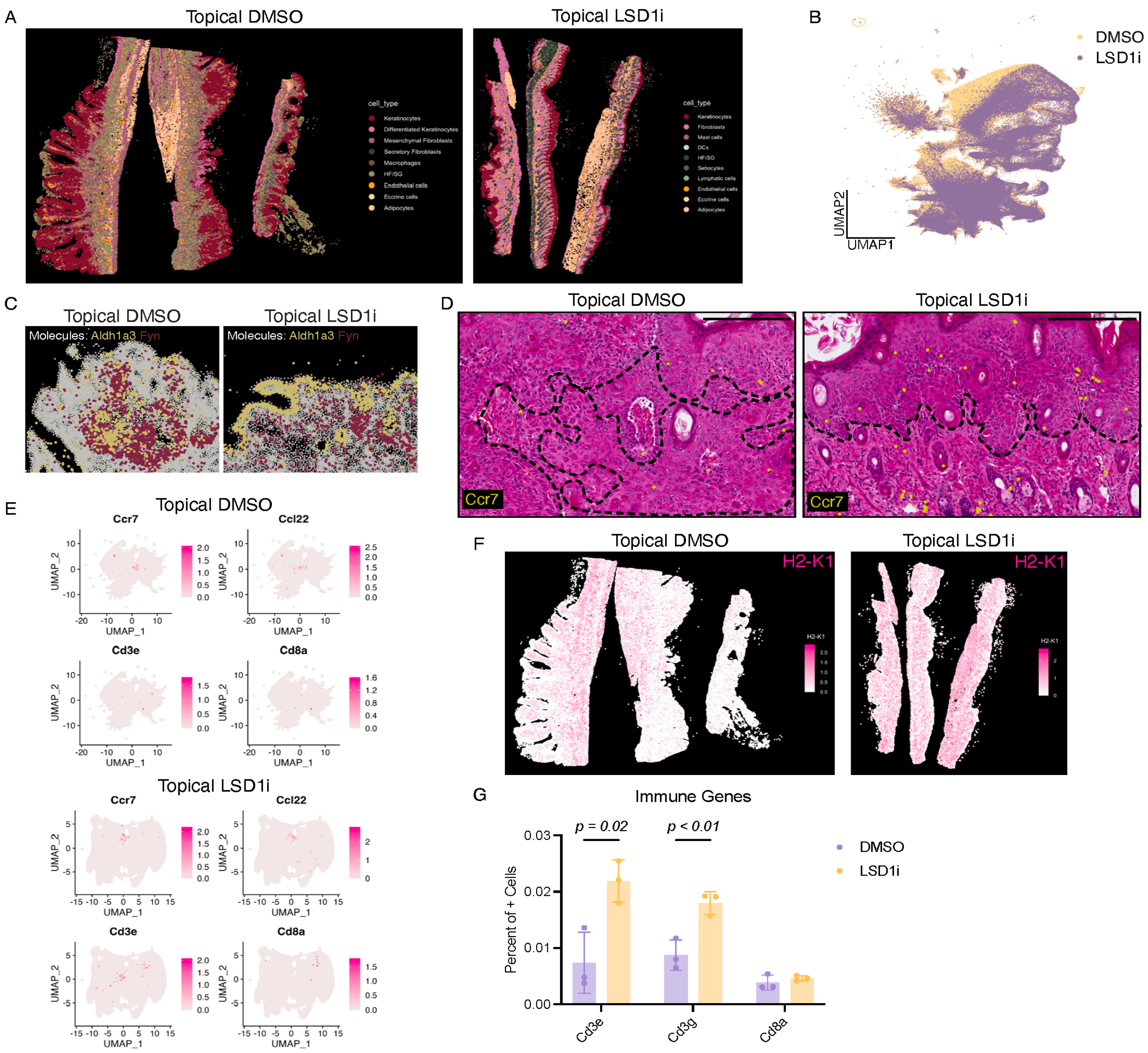


**Fig. S12. LSD1 inhibition facilitates retinoid and immune signaling in skin tumors.** (**A**) Xenium dimplot of cell type clusters on tumor sections from Fyn mice treated with DMSO or LSD1i. (**B**) Merged DMSO and LSD1i Xenium data used for Fig.5, D and F (**C**) Xenium dimplot from Fyn tumors, *Aldh1a3* (yellow) and *Fyn* (red) labeled. (**D**) Xenium H&E from Fyn tumors, *Ccr7* (yellow) labeled. Dotted black line represents approximation of epidermal-dermal junction or tumor area. Scale bar: 200µM. (**E**) UMAP feature plots of DC and T cell genes from Xenium in Fyn tumors. (**F**) Feature plot from Xenium depicting average expression of MHC II gene H2-K1 in Fyn tumors. (**G**) Percent of cells expressing T cell genes calculated from Xenium in topical LSD1i or DMSO treated tumors; One-way ANOVA. All scale bars: 100µM unless indicated. Data represented as mean ± SD. Each dot represents an individual mouse on bar graphs: for (A) to (G), n = 3 mice per condition.

**Table S11. qPCR primers used in this study**

| Primer | Sequence |
|---|---|
| **mActin_F** | TGAAGTGTGACGTGGACATC |
| **mActin_R** | GCAGGAGCAATGATCTTGAT |
| **mAldh1a1_F** | GGAATACCGTGGTTGTCAAGCC |
| **mAldh1a1_R** | CCAGGGACAATGTTTACCACGC |
| **mAldh1a2_F** | CACAAGACACGAGCCCATTGGA |
| **mAldh1a2_R** | GGTTTGATGACCACGGTGTTACC |
| **mAldh1a3_F** | CAGCAATTTCCTCCCATCCG |
| **mAldh1a3_R** | CCTCCTAGCTCCAGTGTGAC |
| **mCcl19_F** | ATGTGAATCACTCTGGCCCAGGAA |
| **mCcl19_R** | AAGCGGCTTTATTGGAAGCTCTGC |
| **mCcl20_F** | GTGGGTTTCACAAGACAGATGGC |
| **mCcl20_R** | CCAGTTCTGCTTTGGATCAGCG |
| **mCcl21_F** | TGAGCTATGTGCAAACCCTGAGGA |
| **mCcl21_R** | TGAGGGCTGTGTCTGTTCAGTTCT |
| **mCcl22_F** | GTGGAAGACAGTATCTGCTGCC |
| **mCcl22_R** | AGGCTTGCGGCAGGATTTTGAG |
| **mCcr7_F** | TCATTGCCGTGGTGGTAGTCTTCA |
| **mCcr7_R** | ATGTTGAGCTGCTTGCTGGTTTCG |
| **mCrabp2_F** | ATGCCTAACTTTTCTGGCAACT |
| **mCrabp2_R** | CCTGTTTGATCTCGACTGCTG |
| **mCsf2_F** | AACCTCCTGGATGACATGCCTG |
| **mCsf2_R** | AAATTGCCCCGTAGACCCTGCT |
| **mIl1a_F** | ACGGCTGAGTTTCAGTGAGACC |
| **mIl1a_R** | CACTCTGGTAGGTGTAAGGTGC |
| **mIl1b_F** | CCTCAAAGGAAAGAATCTATACCTG |
| **mIl1b_R** | CTTGGGATCCACACTCTCC |
| **mRbp2_F** | CTACGACCTGGATTTCACCGTC |
| **mRbp2_R** | ACTGCTTCCAGCCACGGTTCTC |
| **mTslp_F** | GCAAATCGAGGACTGTGAGAGC |
| **mTslp_R** | TGAGGGCTTCTCTTGTTCTCCG |
| **hCCR7_F** | ATTACTACAACCGATCCACCTC |
| **hCCR7_R** | TAGGAGCATGCCACTGAAGA |
| **hCSF2_F** | CACTGCTGCTGAGATGAATGAAA |
| **hCSF2_R** | GTCTGTAGGCAGGTCGGCTC |
| **hIL1b_F** | ATGCACCTGTACGATCACTG |
| **hIL1b_R** | ACAAAGGACATGGAGAACACC |
| **hSNAI2_F** | GGACACACATACAGTGATTATTTCC |
| **hSNAI2_R** | CTTGGACTGTAGTCTTTCCTCTTC |

**Table S12. Antibodies and dilutions used in this study**

| Antibody | Supplier | Catalog # | Host | Dilution IHC | IF | Western | Cut&Run or ChIP |
|---|---|---|---|---|---|---|---|
| **B-ACTIN** | Cell Signaling | 4970S | Rabbit | | | 1:1000 | |
| **CD3** | Abcam | 5690 | Rabbit | | 1:100 | | |
| **CRABP2** | Proteintech | 10225-1 AP | Rabbit | | 1:250 | 1:300 | |
| **Cytokeratin 10** | Abcam | 76318 | Rabbit | | 1:500 | | |
| **Cytokeratin 14** | Abcam | 7800 | Mouse | | 1:500 | | |
| **H3K4me1** | Abcam | 8895 | Rabbit | 1:2000 | 1:1000 | | |
| **H3K4me2** | Abcam | 7766 | Rabbit | 1:750 | | | 1:50 |
| **INVOLUCRIN** | Biolegend | 924401 | Rabbit | | 1:1000 | | |
| **Ki67** | Abcam | 15580 | Rabbit | | 1:500 | | |
| **KLF4** | R&D Systems | AF3158 | Goat | | 1:100 | | |
| **LORICRIN** | Abcam | 85679 | Rabbit | 1:200 | | | |
| **LSD1 (KDM1A)** | Abcam | 17721 | Rabbit | 1:500 | | 1:1000 | 1:50; 2μg |
| **OVOL2** | Thermo Fisher | PA5-115700 | Rabbit | 1:100 | | | |
| **p63** | Biolegend | 619002 | Rabbit | 1:200 | | | |
| **PD1** | Cell Signaling | 846515 | Rabbit | 1:100* | | | |
| **RBP2** | Abcam | 233514 | Rabbit | | 1:80 | 1:500 | |
| **H3K4me3** | Cell Signaling | 9751T | Rabbit | | | | 1:50 |
| **IgG (DA1E)** | Cell Signaling | 66362S | Rabbit | | | | 1:20 |

*30 minute citrate antigen retrieval

**Table S13. Flow cytometry antibodies and dilutions used in this study**

| Fluorochromes | Target | Catalog # | Dilution | Tissue | Panel |
|---|---|---|---|---|---|
| **BUV395** | CD45.2 | BD 564279 | 1:500 | Skin/LN | Panel 1, 2, 3 |
| **Live Dead Blue** | Dead | Thermo Fisher L34962 | 1:2400 | Skin;LN | Panel 1, 2, 3 |
| **BUV737** | CD11c | BD 612797 | 1:500 | Skin/LN | Panel 1, 3 |
| **Pacific Blue** | CD8a | BD 558106 | 1:500 | Skin | Panel 1 |
| **BV510** | Ly6C | Biolegend 128033 | 1:500 | Skin | Panel 1 |
| **BV605** | F4/80 | BD 743281 | 1:500 | Skin | Panel 1 |
| **BV650** | Ly6g | Biolegend 127641 | 1:500 | Skin | Panel 1 |
| **BV711** | IA/E | BD 563414 | 1:500 | Skin | Panel 1 |
| **BV750** | B220 | Biolegend 103261 | 1:500 | Skin | Panel 1, 2, 3 |
| **BV785** | CD11b | Biolegend 101243 | 1:500 | Skin | Panel 1 |
| **PerCP-Cy5.5** | Fcerla | Biolegend 134319 | 1:500 | Skin | Panel 1 |
| **PE-Cy5** | Ter119 | Biolegend 116210 | 1:500 | Skin | Panel 1, 2, 3 |
| **PE-Cy7** | CD4 | Biolegend 100422 | 1:500 | Skin | Panel 1 |
| **APC** | CD207 | Biolegend 144205 | 1:500 | Skin | Panel 1 |
| **APC-Cy7** | SiglecF | Biolegend 155532 | 1:500 | Skin | Panel 1 |
| **PE-Cy7** | IL-17 | Thermo 25-7177-80 | 1:500 | Skin | Cytokine |
| **PE** | RARγ (G1) | Santa Cruz 7387-PE | 1:500 | Skin | Panel 2 |
| **BV421** | CD3 | BD 564008 | 1:500 | Skin/LN | Panel 2, 3 |
| **Pacific Blue** | IA/E | Biolegend 107619 | 1:500 | LN | Panel 3 |
| **BV570** | CD11b | Biolegend 101233 | 1:500 | LN | Panel 3 |
| **BV650** | CD8a | Biolegend 100741 | 1:500 | Skin/LN | Panel 2, 3 |
| **AF700** | CD4 | ThermoFisher 56-0042-80 | 1:500 | Skin/LN | Panel 2, 3 |
| **BV605** | CTLA4 | Biolegend 106323 | 1:200 | Skin/LN | Panel 2, 3 |
| **PE-Cy7** | PD-1 | Biolegend 109110 | 1:500 | Skin/LN | Panel 2, 3 |
| **PE/Dazzle 594** | PD-L2 | Biolegend 107215 | 1:500 | Skin/LN | Panel 2, 3 |
| **BV785** | Tbet | Biolegend 644825 | 1:200 | Skin/LN | Panel 2, 3 |
| **APC** | Ki67 | Biolegend 652421 | 1:200 | Skin/LN | Panel 2, 3 |
| **PE** | ICOS | Biolegend 107706 | 1:500 | LN | Panel 3 |
| **PE/Cy7** | CD8 | Biolegend 100722 | 1:100 | Spleen | Panel 4 |
| **L/D aqua** | Viability | Invitrogen L34957 | 1:600 | Spleen/Tumor | Panel 4,5 |
| **AF700** | CD45 | Biolegend 103128 | 1:100 | Spleen/Tumor | Panel 4,5 |
| **BV650** | CD4 | Biolegend 100555 | 1:100 | Spleen/Tumor | Panel 4,5 |
| **eFluor 450** | CD11b | ThermoFisher 48-0112-82 | 1:160 | Tumor | Panel 5 |
| **PE-CF594** | CD11c | Biolegend 117347 | 1:80 | Tumor | Panel 5 |
| **AF488** | Ly6C | Biolegend 128021 | 1:200 | Tumor | Panel 5 |
| **PE-Cy5** | Ly6g | Biolegend 127672 | 1:80 | Tumor | Panel 5 |
| **APC-Cy7** | MHC II | Biolegend 116426 | 1:80 | Tumor | Panel 5 |
