# Supplementary material for: Inhibiting LSD1 unlocks retinoid AP-1 programming to activate epithelial immunity and skin tumor suppression
Source: J Clin Invest. 2026 Mar 12;136(8):e189044. doi: 10.1172/JCI189044 (PMC13078892; doi:10.1172/JCI189044)

Supplementary - Unedited Gels

A Full unedited gel for Fig. S3A

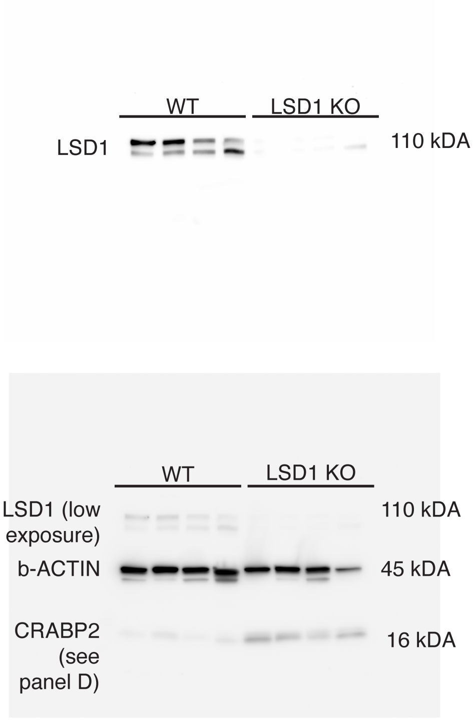

B Full unedited gel for Fig. S3G

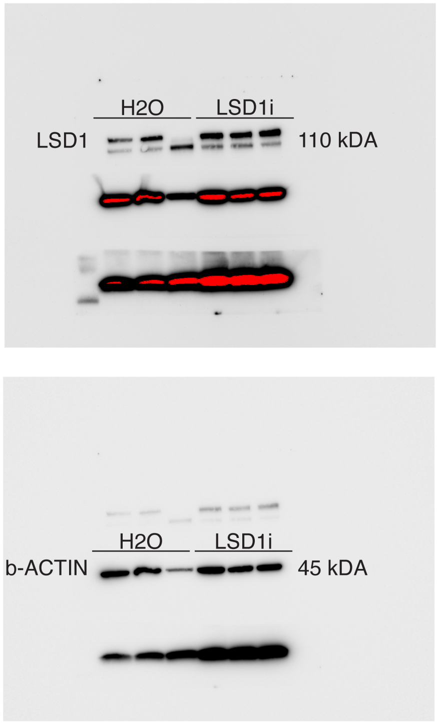

C Full unedited gel for Fig. S4G

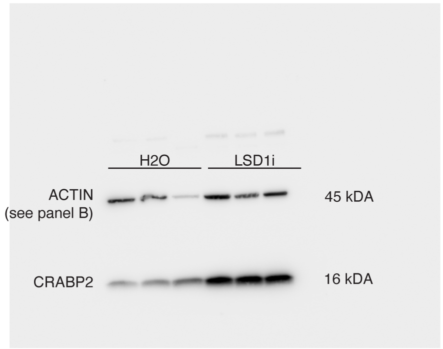

D Full unedited gel for Fig. S6C (top)

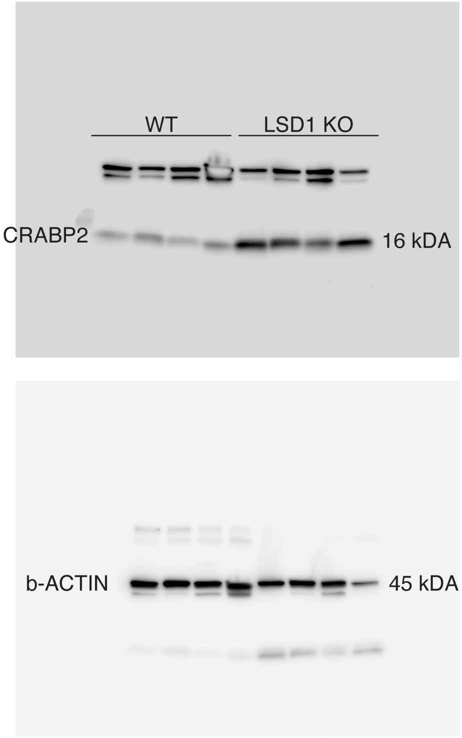

E Full unedited gel for Fig. S6C (bottom)

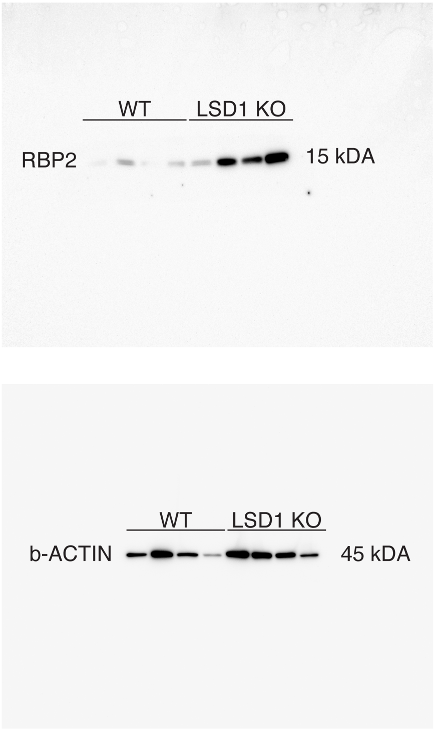

Supplement: Unedited blot and gel images [file jci-136-189044-s140.pdf]
